# Parliament2: Accurate structural variant calling at scale

**DOI:** 10.1093/gigascience/giaa145

**Published:** 2020-12-21

**Authors:** Samantha Zarate, Andrew Carroll, Medhat Mahmoud, Olga Krasheninina, Goo Jun, William J Salerno, Michael C Schatz, Eric Boerwinkle, Richard A Gibbs, Fritz J Sedlazeck

**Affiliations:** DNAnexus, 1975 W El Camino Real #204, Mountain View, CA 94040, USA; Department of Computer Science, 3400 N. Charles St. Johns Hopkins University, Baltimore, MD 21218, USA; DNAnexus, 1975 W El Camino Real #204, Mountain View, CA 94040, USA; Human Genome Sequencing Center, One Baylor Plaza, Baylor College of Medicine, Houston, TX 77030, USA; Human Genome Sequencing Center, One Baylor Plaza, Baylor College of Medicine, Houston, TX 77030, USA; Human Genetics Center, 1200 Pressler Street, University of Texas Health Science Center at Houston, Houston, TX 77040, USA; Human Genome Sequencing Center, One Baylor Plaza, Baylor College of Medicine, Houston, TX 77030, USA; Department of Computer Science, 3400 N. Charles St. Johns Hopkins University, Baltimore, MD 21218, USA; Human Genome Sequencing Center, One Baylor Plaza, Baylor College of Medicine, Houston, TX 77030, USA; Human Genetics Center, 1200 Pressler Street, University of Texas Health Science Center at Houston, Houston, TX 77040, USA; Human Genome Sequencing Center, One Baylor Plaza, Baylor College of Medicine, Houston, TX 77030, USA; Human Genome Sequencing Center, One Baylor Plaza, Baylor College of Medicine, Houston, TX 77030, USA

**Keywords:** structural variation, next-generation sequencing, high-throughput sequencing

## Abstract

**Background:**

Structural variants (SVs) are critical contributors to genetic diversity and genomic disease. To predict the phenotypic impact of SVs, there is a need for better estimates of both the occurrence and frequency of SVs, preferably from large, ethnically diverse cohorts. Thus, the current standard approach requires the use of short paired-end reads, which remain challenging to detect, especially at the scale of hundreds to thousands of samples.

**Findings:**

We present Parliament2, a consensus SV framework that leverages multiple best-in-class methods to identify high-quality SVs from short-read DNA sequence data at scale. Parliament2 incorporates pre-installed SV callers that are optimized for efficient execution in parallel to reduce the overall runtime and costs. We demonstrate the accuracy of Parliament2 when applied to data from NovaSeq and HiSeq X platforms with the Genome in a Bottle (GIAB) SV call set across all size classes. The reported quality score per SV is calibrated across different SV types and size classes. Parliament2 has the highest F1 score (74.27%) measured across the independent gold standard from GIAB. We illustrate the compute performance by processing all 1000 Genomes samples (2,691 samples) in <1 day on GRCH38. Parliament2 improves the runtime performance of individual methods and is open source (https://github.com/slzarate/parliament2), and a Docker image, as well as a WDL implementation, is available.

**Conclusion:**

Parliament2 provides both a highly accurate single-sample SV call set from short-read DNA sequence data and enables cost-efficient application over cloud or cluster environments, processing thousands of samples.

## Findings

Structural variants (SVs) comprise a broad class of genomic alterations, typically defined as events 50 bp or larger, including deletions, duplications, insertions, inversions, and translocations. SVs are critical to fully understanding evolutionary processes, gene expression, and genomic diseases such as Mendelian disorders and cancer [1–3]. Accurate SV detection is limited by the inherent problem that SVs are generally larger than the short reads that compose the majority of sequencing data. Therefore, SVs are usually inferred by including split-read mapping, soft-clipped reads, changes in the distance between and orientation of read pairs, coverage depth variations, and alterations in the heterozygosity of a region [3, 4]. Even best-in-class methods can fail to capture the majority of SVs (30–70% sensitivity) and often return a high false discovery rate, especially for insertion and inversion events [5, 6].

Commonly used SV detection methods, such as Breakdancer [7], CNVnator [8], Crest [9], Delly [10], Lumpy [11], Manta [11, 12], and Pindel [13], rely on heuristic approaches leveraging some or most of the mapped-read signals. This diversity of approaches also results in performance heterogeneity across SV types and size regimes, as well as varied compute requirements. The differences in approaches also allow for ensemble optimization. Two methods, MetaSV [14] and Parliament [15], use a 3-step overlap-merge-validate strategy to combine results of multiple callers into a high-quality consensus set. Both MetaSV and Parliament use an assembly-based method for the validation step, which, while accurate, is computationally intensive and limits the maximum size of events [15]. Because MetaSV and Parliament start from existing SV calls, they place the burden of installing and running individual SV callers on the user. Furthermore, the computational requirements present additional challenges to at-scale execution for large sample sets.

Here we present Parliament2, a scalable SV caller optimized for cloud-based analysis with high precision and recall designed for single-sample analysis and large cohort aggregation. Parliament2 executes any combination of Breakdancer, Breakseq, CNVnator, Delly, Lumpy, and Manta to generate candidate SV events; uses SURVIVOR [16] to overlap these calls into consensus SV candidates; validates these calls using SVTyper [16, 17]; and for each event assigns a quality value derived from the SV size, type, and combination of supporting methods. Parliament2 reports multiple SV types including deletions, duplications, insertions, inversions, and translocations. Computational efficiency is achieved via multiple parallelization strategies that execute callers simultaneously, taking advantage of the complementary requirements in CPU, disk I/O, and RAM. This parallelization speeds up the individual methods and thus allows Parliament2 a faster execution time than running each program on its own. A 16-core machine can process a 35× whole-genome sequence (WGS) sample in 2–5 hours. Parliament2 is tunable in terms of recall and precision, meeting the needs of multiple experimental designs, such as maximal sensitivity in research settings and clinical-grade precision for diagnostics. Parliament2 has been tested across multiple platforms and optionally provides PDF images for manual curation using SVVIZ [18].

Parliament2 is open source and available as a code base [19], a DNAnexus app, and a Docker image that can be used to easily run any combination of individual callers [19].

### Accuracy assessments for Parliament2 based on real data

**Figure 1:**
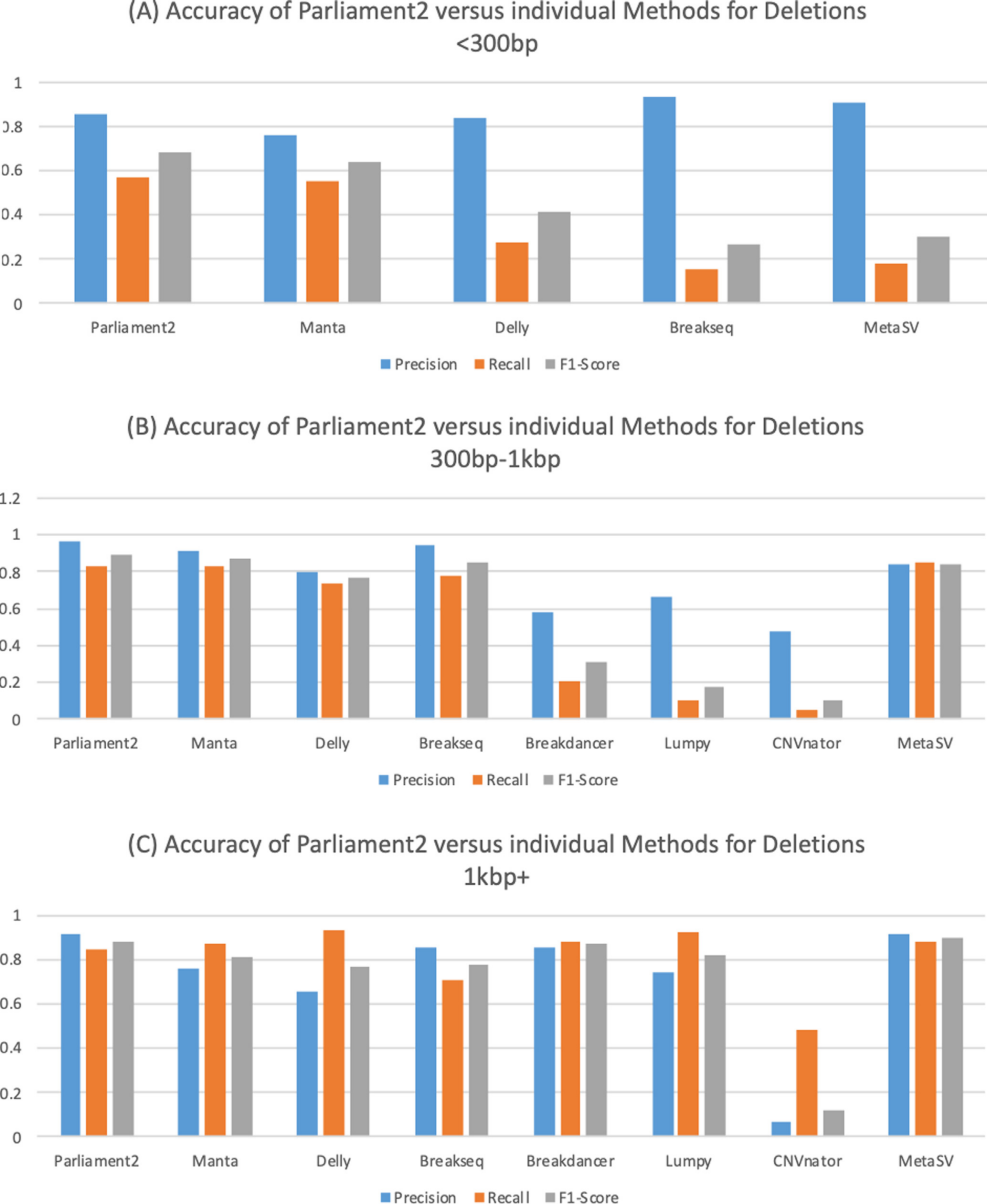
Accuracy comparison for Parliament2 based on GIAB v0.6 deletion call set for different size regimes of deletions: (A) <300 bp, (B) between 300 bp and 1 kb, and (C) >1 kb. The order of methods in each graph is sorted such that methods with higher F1 scores are located to the left. The efficacies of individual methods vary between size ranges.

We assessed the performance of Parliament2 in terms of precision (1 − false discovery rate), recall (true-positive rate), and runtime compared to other short-read SV methods (using their default or otherwise suggested parameters) based on the Genome in a Bottle (GIAB) v0.6 SV candidate truth set [20] and using the suggested Truvari software [21] for comparing SV calls >50 bp [22]. The GIAB SV truth set is based on HG002, a male Ashkenazi Jewish sample using multiple technologies and manual vetting of the SV. Parliament2 ran in 3.43 hours (wall time) for this sample on a 16-core machine from a 35× coverage BAM aligned to the hs37d5 reference genome. While Parliament2 can infer multiple SV types, the current GIAB call set largely comprises insertion and deletion events. Apart from other SV callers, we also benchmarked MetaSV, which also leverages multiple SV callers together. Owing to the complexity of MetaSV, we used the results submitted by their authors to GIAB. Figure 1**A** shows the results for small deletions (50–300 bp) (see Supplementary Table S1 for details). The vast majority of the GIAB call set includes 32,520 (86.92%) deletions of this size range, highlighting its importance in detecting these events. We obtained only deletion calls from Manta, Delly, and Breakseq for this size category. Parliament2 had the highest recall rate (56.54%) while having the third-highest precision (85.17%). Only Breakseq (93.20%) and Meta-SV (90.84%) had a higher precision, likely due to their having the lowest recall rates, calling only 15.69% and 18.04% of the deletions, respectively. Thus, Parliament2 (67.96%) had the highest F1 score (i.e., harmonic mean of precision and recall), followed by Manta (64.00%). Fig. 1**B** shows the performance of the different SV calling methods over the 3,278 (8.76%) mid-size deletions (300–1,000 bp) (see Supplementary Table S1 for details). Parliament2 had the second-highest recall (83.20%) and the highest precision (96.49%). Only MetaSV had a marginally higher recall (83.81%). Again, Parliament2 showed the highest F1 score (89.35%), followed by Manta (86.65%). For deletions >1 kb (Fig. 1**C**; see Supplementary Table S1 for details), which are only 1,614 (4.31%) of the gold standard, MetaSV, showed the highest F1 score (89.83%), closely followed by Parliament2 (87.89%), both driven by their high precision scores of 91.92% and 91.59%, respectively. Across all size regimes for deletions, Parliament2 achieved by far the highest F1 score (mean: 81.73%) followed by Manta (77.31%), MetaSV (68.06%), Delly (65.20%), Breakseq (63.28%), Breakdancer (58.78%), Lumpy (49.96%), and CNVnator (11.12%).

We further assessed the recall and precision across insertions for Parliament2 over the HG002 sample. Across all callers, Parliament2 yielded a high precision score (94.13%) and a recall score consistent with GIAB's incorporation of long-read technologies (19.21%).

To avoid a biased benchmarking, we further benchmarked Parliament2 against 3 assembly-based SV call sets [23] from nonwhites to highlight Parliament2’s versatility. Supplementary Fig. S2 shows the results split for deletions and insertions because these are the only SVs previously reported. Parliament2 again achieves the highest recall and precision for insertions and deletions. Nevertheless, as expected, the recall for insertions is reduced compared to deletions given the limitations of short-read–based insertion detection.

### Compute efficiency

Runtime and computational efficiency are essential to scalability and cost reduction. The SV callers used by Parliament2 fall into 3 parallelization classes: native multi-threading (Breakseq, Manta), native parallelization by chromosome (CNVnator, Breakdancer), and those lacking either (Delly, Lumpy). Upon execution, Parliament2 immediately executes Breakseq and Manta with multiple threads, splits the input BAM by chromosome, and initiates runs on the remaining callers. For the 35× HG002 BAM, this strategy reduced the runtime for Lumpy from 6.45 to 0.45 hours and for Delly from 8.52 to 0.67 hours on a 16-core machine.

**Figure 2:**
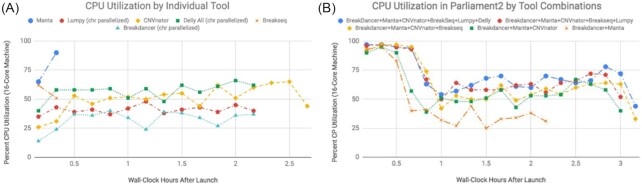
Concurrent execution of multiple tools in Parliament2 increases resource utilization. (A) Percent of total CPU utilization on a 16-core machine executing Parliament2 and running only an individual tool. Each line terminates when the program finishes executing. (B) Resource utilization when running combinations of methods simultaneously within Parliament2.

The parallelization across multiple programs leads to a reduction in runtime by achieving higher overall machine utilization of resources (Fig. 2). In local and cloud environments, this optimization translates to reductions of cost, CPU utilization, and wall time.

### Consensus quality scores

One oft-discussed problem for short-read-based SV calling is low sensitivity and high false discovery rates [5, 6]. This challenge is exacerbated by the variety of SV types and sizes and the applicability of various methods to each SV class. The different performances of individual methods (see above) highlight the potential of a consensus approach stratified by size and event type. Without such a distinction, accuracy assessments are dominated by the more numerous small events, potentially under-reporting rare but impactful gene-sized events.

**Figure 3:**
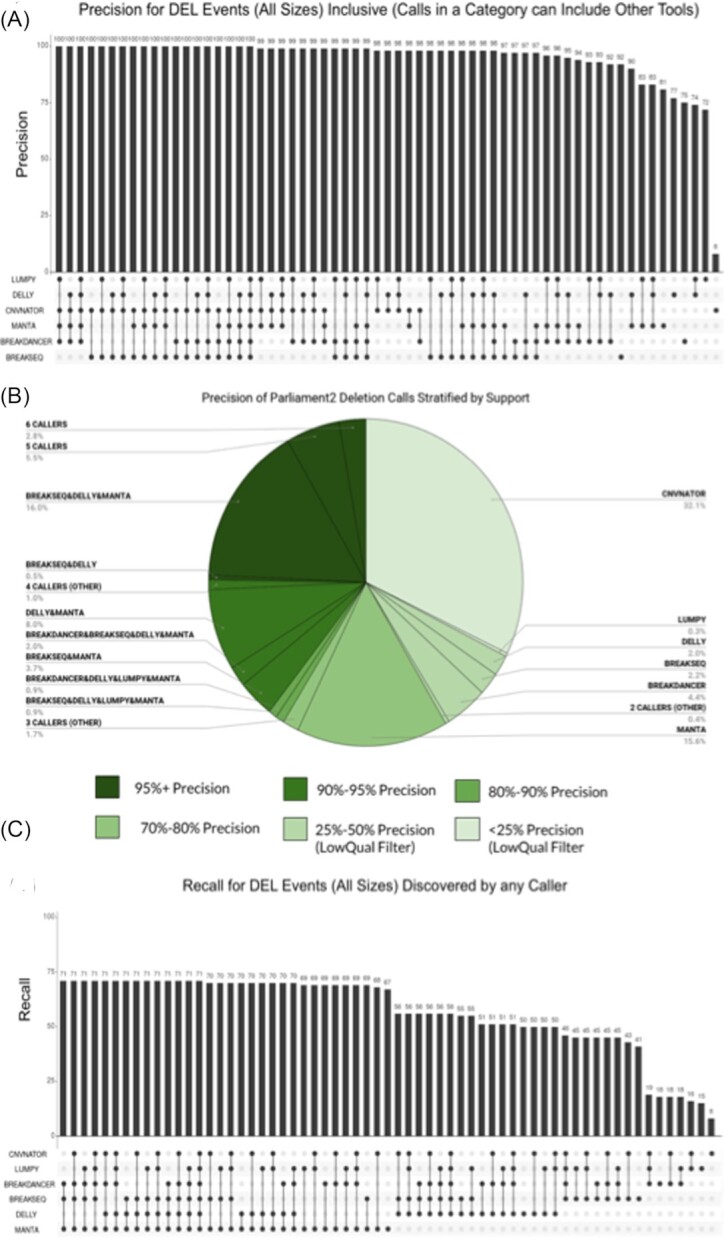
Assessment of constituent SV-calling methods based on the deletion call set from GIAB v0.6. (A) Measured precision for the different method combinations. The precision ranges from 5% (CNVnator) up to 100% for various combinations. (B) Contributions of the individual SV callers and their combinations to the total number of calls (percent label) and their relative precision (color-coded by shade of green). (C) Measured recall for individual methods and their combinations ranging from 8% (CNVnator) to 71% for various combinations.

We analyzed the contribution of each Parliament2 caller to the overall precision [22]. Fig. 3**A** describes how each combination of SV methods contributes to recall performance. The precision of SV calls obtained by a single individual method ranges from 8% with CNVnator to 91% for Breakseq. However, when an SV call is supported by multiple methods, precision can reach 100% independent of the size regime (Fig. 3A and **B**). The combination of CNVnator and BreakSeq is the minimum set of SV callers to reach 100% precision. Although CNVnator has the lowest precision performance (8%), it is included in every set that reaches a 100% recall rate. Thus, while deletions discovered only by CNVnator have low precision, a deletion call from CNVnator and ≥1 other method provides high precision. While only a few methods (Breakseq, Manta, and Delly) detect insertions, they are generally precise (98–100%). Supplementary Fig. S1 shows the precision of the individual SV caller and their combinations for insertions.

Figure 3**C** details the recall rates of individual SV callers and their combinations. The highest recall rate (71%) is achieved by a combination of multiple callers. This value is surprisingly high given that the truth set includes data from multiple long-read technologies and SVs that were only obtained by long-read sequencing and assembly. Manta is included in all of the combinations that reached a high recall value for deletions. For insertions, the overall recall is drastically reduced to 17% using a combination of Manta (15%), Delly (3%), and BreakSeq (2%) (Supplementary Fig. S1).

On the basis of these observations, we generated a ruleset based on GIAB deletion and insertion calls using the supporting callers, type of event, and size of event (for deletions), assuming that the individual SV callers show similar metrics in other types of SVs. This ruleset is then applied to assign quality values to each of the reported SV calls. Parliament2 expresses the call quality as a Phred-encoded value within the final consensus VCF. These scores are based on the precision results from GIAB for each combination of supporting callers, the type of the event (deletion or insertion), and the size category of the event (50–300 bp, 300 to 1,000 bp, >1 kb). This quality value allows investigators to set thresholds to achieve the trade-off between precision and recall that is desired for their use cases or to prioritize events on the basis of how likely they are to be true events. Supplementary Fig. S1 shows the quality value based on caller support and the SV's type and size. These quality values enable Parliament2 to obtain a balanced performance for recall rate and precision, resulting in the highest F1 scores (Fig. 1) across multiple size regimes. The same ruleset is also applied to other SV types for which we lacked GIAB benchmark data (e.g., inversions).

### Interplatform concordance

**Figure 4.**
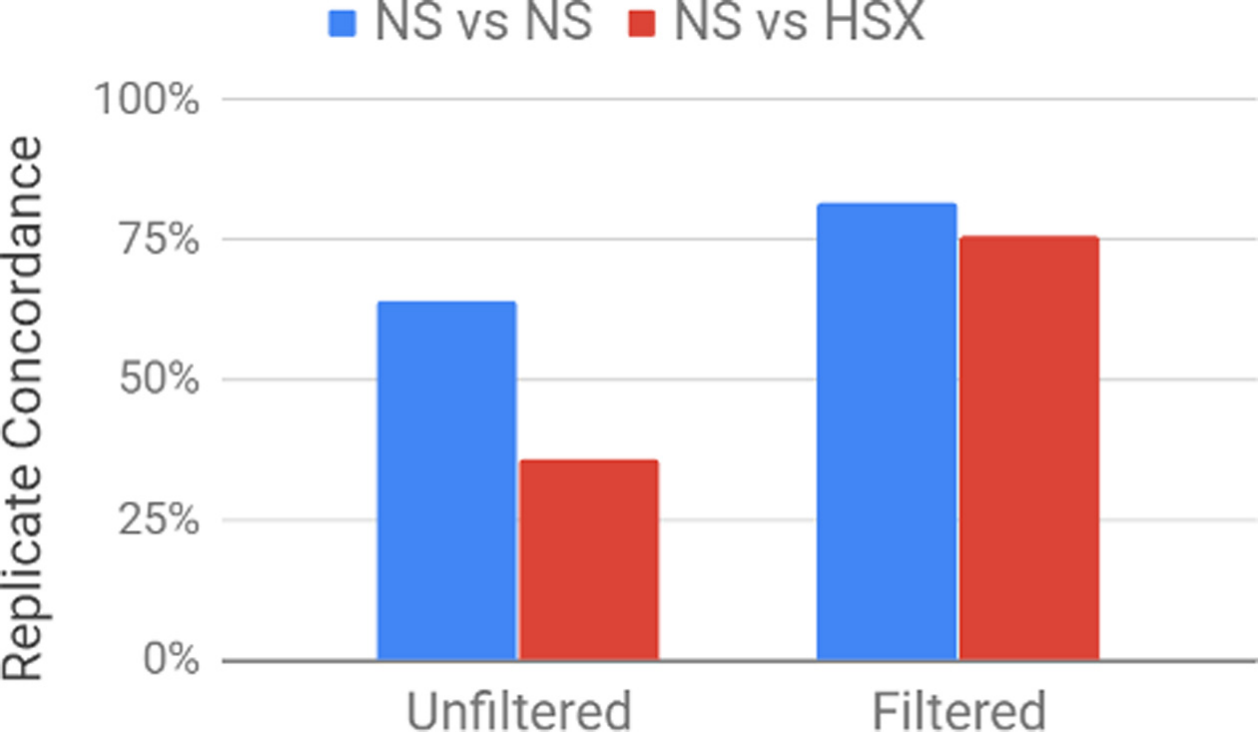
Parliament2 HG002 concordance across NovaSeq (NS) and HiSeqX (HSX) before and after quality filtering.

Large collaborative projects aggregate heterogeneous data across different sequencing centers, chemistry versions, and short-read platforms (e.g., HiSeq X and NovaSeq). Given the inferential nature of SV detection from short reads, SV methods are particularly susceptible to batch effects. Therefore, we have characterized Parliament2 using HiSeq X and NovaSeq sequencing runs, including the aforementioned HiSeq X data and 4 NovaSeq HG002 replicates each downsampled to 35× coverage and mapped to the hs37d5 reference. These 35× NovaSeq replicates showed similar precision (83.0%) and recall (69.35%) compared to the HiSeq X (81.7% and 70.7%, respectively). Increasing coverage to 50× for all samples across both platforms changed these values by <5% (see Supplementary Table S2 for details), indicating the robustness of evaluating both platforms at 35×. The unfiltered concordance values, corresponding to all raw Parliament2 consensus calls, indicate low interplatform consistency, which would likely drive batch effects in mixed-platform sample sets (Fig. 4). After filtering for Parliament2 events with a quality value >3, inter- and intraplatform concordances increase to similar levels, suggesting both an increase in quality and mitigation of platform batch effects (Fig. 4).

### 1000 Genomes Project SVs for GRCh38

The 1000 Genomes Project (1KGP) is a valuable resource of high-confidence SV calls across a large sample set (2,691 samples) mapped to GRCh37. However, since the introduction of GRCh38 [24], many large-scale whole-genome programs (e.g., TOPMed, All of Us) have adopted this standard. To demonstrate the scalability of Parliament2 for large datasets and to create a community resource, we applied Parliament2 to the 2,691 1KGP WGS samples mapped to GRCh38 [24]. Although the 1KGP samples have been remapped to GRCh38 [24, 25], we are not aware of a comprehensive set of SVs on these data and reference sets.

**Figure 5.**
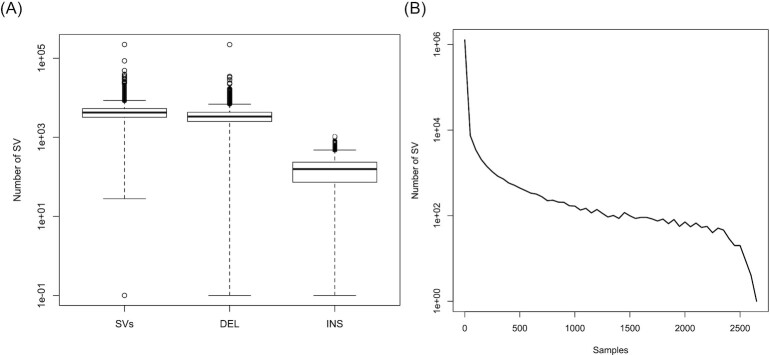
Population distribution of SV calls produced by Parliament2 for the 1000Genomes Phase3. (A) Number of SVs, deletions, and insertions across the 2,506 samples. Each type shows the the minimum, first quartile, median, third quartile, and maximum per individual. (B) Allele frequency across all SVs at log scale showing an expected distribution of a high number of rare SVs and very few common SVs or fixed SVs.

The computational requirements were modest in comparison to other familiar applications, and the entire SV calling was completed in 1 day of wall-clock time, using only 63,720 CPU-hours (on average 24 core-hours per sample). For reference, that amount of compute is approximately equivalent to running GATK4 on 220 WGS samples at 35× coverage. This effort created SV calls for each of Breakdancer, CNVnator, Delly, Lumpy, and Manta, as well as SVTyped files of each and consensus Parliament2 calls. Figure 5 shows the results for these call sets [22].

Figure 5**A** shows the distribution of SV inferred per sample across all 1KGP samples. In total, there are 88,404 deletions >50 bp discovered in this set and 30,479 inversion events. There were only 619 insertions discovered, possibly reflecting that it is more difficult to detect an insertion in these low-coverage data. The number of calls per sample was generally lower than observed for the high-coverage WGS samples investigated in the prior benchmarks (Fig.5A). Additionally, for certain samples, some of the callers did not generate any output, possibly due to low sequence coverage of the samples. Nevertheless, the allele frequency profile looks as expected (Fig. 5**B**) because it reassembles a high amount of private SV vs a much lower amount of common SV. In addition, we observed only minor fixated alleles as the samples span a large set of different populations. These SV calls will provide a resource to understand SVs called on GRCh38 relative to the multiple ethnicities captured in 1KGP and to understand how each of these tools interacts with lower coverage data.

### Interoperability of Parliament2 using WDL

A newer version of Parliament2, which uses the same tools and principles discussed earlier, is open-source and available as a code base [19]. This version increases the interoperability of Parliament2. Instead of implementing all tools on a single Docker image, this workflow uses the Workflow Development Language (WDL) to run each parallelized SV caller as a task, with separate Docker images implemented for each tool. This can then be imported to different cloud platforms, including DNAnexus and AnVIL/Terra [26], as well as any environment configured to run Cromwell, such as high-performance computing clusters. As a result, this version of Parliament2 enables a better adoption to other infrastructures and is more modularly implemented.

This WDL workflow version runs in 4.52 hours (wall time) compared to the main version (3.43 hours) on the same 35× coverage BAM aligned to the hs37d5 reference genome used for benchmarking in previous sections. The increased runtime is likely due to both the increased I/O required for spinning up different machines for each task and the fact that these machines had <16 cores.

Furthermore, the WDL version of Parliament2 upgrades several tools. As a result of these upgrades, the SV call set produced is modestly different than that generated by the original version of Parliament2 benchmarked here, although the overlap is high (87.37% of the WDL output overlaps with the original). Among insertions and deletions, which we benchmark above, overlap is 84.87% for insertions, 90.95% for deletions, and 90.23% for both insertions and deletions combined. We inspected the differences between the call sets and found that they were chiefly due to borderline calls that were just above or below the thresholds of the tools used. This version also integrates Jasmine [27] as an alternative to SURVIVOR for merging SVs.

In this article, we present Parliament2 for identifying SVs at scale for short-read datasets. The Parliament2 optimized consensus approach addresses the accuracy and compute challenges of calling SVs from short-read sequence data at scale. Leveraging consensus calling for event discovery and quality assessment, Parliament2 achieves a higher overall accuracy (F1 score against GIAB HG002 SVs) than any constituent method without compromising efficiency, providing robust SV calling across multiple platforms. Parliament2 is unique in its capability to identify multiple classes of SVs in an easily scaled manner, enabling efficient computation on a single sample (∼3 hours) or on thousands of samples. Within 1 day of Parliament2 compute, we have generated the first comprehensive SV set for the 1KGP samples on GRCh38, a publicly available resource.

Parliament2 is specialized relative to MetaSV in 2 key ways. First, Parliament2 is optimized for scalability, not requiring an expert user to launch multiple SV callers, the results of which need to be combined later (e.g., SURVIVOR, MetaSV). This leads to a faster and more efficient execution over thousands of samples. Parliament2 pre-installs these programs, configures them to speed up the processing, and utilizes a trained quality value to provide extra information about the reliability of the SV calls. Second, MetaSV does not provide a full workflow and includes costly assembly steps that result in high computational costs over multiple samples. Nevertheless, these enable MetaSV to report sequence-resolved insertion calls, while Parliament2 can only produce the sequence resolution if the individual method that called the event produced it. Still, this complicates the execution of MetaSV over multiple hundred to thousands of samples required for larger cohorts.

SV calling accuracy, however, remains an open challenge. F1 scores for best-in-class small variant callers routinely exceed 99%, and even higher standards are required for clinical reporting. As SV methods improve, the Parliament2 infrastructure can be easily adapted to incorporate new methods (e.g., graph-based references and rapid local assembly) and SV callers, especially those that target specific SV types such as mobile element insertions and variable nucleotide tandem repeats, to determine the optimal consensus strategy. Such improvement will be accelerated by broader and deeper high-confidence SVs from long-range data across more samples and ethnicities against which SV methods such as Parliament2 can be trained.

## Methods

### Parliament2 implementation

The code for Parliament2 is available at a GitHub repository [19] with an open source (Apache-2.0) license at the 1.0.7 version (commit 97517b1a22104a3e0a0966a79c3b5556fde8a89d). Execution of Parliament2 done by running v1.0.7 of the Parliament2 DNAnexus app (app-FJ8Fj88054JxXFygKvFqQ39j), which is publicly available to run by any user on DNAnexus. This app runs a Docker image built directly from the GitHub repository, which is available on DockerHub. Executions of the app with user-provided input for tool combinations specify the parameter flags to the Docker image to include or exclude the desired tools.

### Parliament2 implementation (WDL)

The WDL version of Parliament2 is available at a GitHub repository [19] with an open-source (Apache-2.0) license at the 0.0.1 version (commit ed86345740f029093365f8a3b0d99f9cb153c9ed). For these tests, the execution of Parliament2 was done by importing this version of the WDL file into DNAnexus, which automatically converts the WDL file into a native workflow. This WDL file specifies Docker image versions, which allow for this code to be easily replicated. The Docker images are available on DockerHub and are built using the code available on the GitHub repository.

### Input WGS data used for timing and accuracy benchmarks

Timing statistics and resource utilization were determined by executing the Parliament2 app on a 35× WGS sample for HG002 that was made by randomly downsampling the 50× PCR-Free HG002 HiSeqX sample generated for the Challenge set of the PrecisionFDA Truth Challenge.

### Timing for individual tools and Parliament2 combinations

All timing calculations are run on a c3.4xlarge AWS instance (16-core, 30 GB RAM, 320 GB disk). To calculate the runtime and resource utilization of individual components, the Parliament2 app was launched with the desired tool or tool combinations. DNAnexus apps write an entry of machine resources (CPU percent, RAM, and disk utilization) every 10 minutes to a job log that also contains the stdout and stderr outputs for job execution. All info log entries of this after the stderr line for program execution up until the SVTyper step (which indicates completion of all jobs) were taken to determine the resource plots over time. The logs for these jobs are available on GitHub [28].

### Accuracy comparisons

Accuracy comparisons are performed using Truvari [21] with the following execution: truvari.py -b GIAB_DEL0.6.vcf.gz -c <parliament_output> -o <output_directory –passonly –includebed GIAB_0.6.bed –pctsim = 0 -r 2000 –giabreport

To determine accuracy for size ranges, -s <lower_size> and -S <upper_size> were used. The deletion truth set was taken by extracting SVTYPE = DEL from the v0.6 truth set. The insertion truth set was taken similarly by extracting SVTYPE = INS.

The GIAB data for v0.6 truth set can be downloaded from [29].

### Tool versions

The individual tools that comprise Parliament2 run the following versions of each program:

Breakdancer: v1.4.3

https://github.com/genome/breakdancer/releases/tag/v1.4.3

BreakSeq2: v2.2

http://bioinform.github.io/breakseq2/

CNVnator: v0.3.3

https://github.com/abyzovlab/CNVnator/commit/de012f2bccfd4e11e84cf685b19fc138115f2d0d

Delly: v0.7.2

https://github.com/dellytools/delly/releases/tag/v0.7.2

Lumpy: v0.2.13

https://github.com/arq5x/lumpy-sv/commit/f466f61e02680796192b055e4c084fbb23dcc692

Manta: v1.4.0

https://anaconda.org/bioconda/manta

SURVIVOR: v1.0.3

https://github.com/fritzsedlazeck/SURVIVOR/commit/7c7731d71fa1cba017f470895fb3ef55f2812067

SVTyper: v0.7.0

https://github.com/hall-lab/svtyper/commit/5fc30763fd3025793ee712a563de800c010f6bea

Svviz: v1.5.2

https://github.com/svviz/svviz/commit/84acefa13bf0d4ad6e7e0f1d058aed6f16681142

The individual tools that comprise the WDL version of Parliament2 run the following versions of each program:

Breakdancer: v1.4.3

https://github.com/genome/breakdancer/releases/tag/v1.4.3

BreakSeq2: v2.2

https://anaconda.org/bioconda/breakseq2/

CNVnator: v0.3.3

https://github.com/abyzovlab/CNVnator/releases/tag/v0.4.1

Delly: v0.8.3

https://anaconda.org/bioconda/delly

Lumpy: v0.3.0

https://anaconda.org/bioconda/lumpy-sv

Manta: v1.4.0

https://anaconda.org/bioconda/manta

SURVIVOR: v1.0.7

https://github.com/fritzsedlazeck/SURVIVOR/commit/1d1d33b016dbf818b1678a27dee3d3de7f0fda0b

JASMINE: v1.0.6

https://github.com/mkirsche/Jasmine/releases/tag/v1.0.6

SVTyper: v0.7.0

https://anaconda.org/bioconda/svtyper

Svviz: v1.6.2

https://anaconda.org/bioconda/svviz

## Availability of Supporting Source Code and Requirements

Project name: Parliament2

Project home page: https://github.com/slzarate/parliament2

Operating system: Linux

Programming language: Bash/Python/C++

Other requirements: Docker

License: Apache-2.0

Biotools ID: parliament2

RRID: SCR_019187

An archival copy of the GitHub repository is also available via the *GigaScience* database GigaDB [22].

## Data Availability

All benchmark results of all the programs are publicly available: https://github.com/slzarate/parliament2/tree/master/benchmarking_data/hg002_benchmarks

1KGP download links for the following resources are as follows:

A project-level VCF of all PASS and unfiltered variants in any sample:

https://github.com/slzarate/parliament2/tree/master/benchmarking_data/1000_genomes

The VCF output of Parliament2:

https://github.com/slzarate/parliament2/tree/master/benchmarking_data/hg002_benchmarks

The individual caller files for Breakdancer, BreakseqCNVnator, Delly, Lumpy, and Manta are available at: https://github.com/slzarate/parliament2/tree/master/benchmarking_data/hg002_benchmarks/sv_caller_outputs

## Additional Files

Supplementary Figure S1: Quality values assigned by Parliament2 to SV events of various types, sizes, and support. Parliament2 assigns a quality value to each event based on the precision observed in comparisons with the GIAB v0.6 truth set. In the above figure, the event type (deletion or insertion) and size determine the color code. The maximum QV assigned is 40, even if the precision of the subset is higher. Only categories with more than two calls are included.

Supplementary Figure S2: Benchmark comparison of Parliament2 on non caucasian samples. We have benchmarked Parliament2 SV calls (insertion and deletions) across three non-caucasian samples (HG00514, HG00733, NA19240) that have been previously characterized by de novo assembly including multiple sequencing technologies. Overall Parliament2 shows a high concordance with the deletion calls to Chaisson et. al. with only very few calls that are private to Parliament2. For insertions, however, the recall ability is reduced due to limitations from the short reads.

Supplementary Table S1: Summary over GIAB evaluation

Supplementary Table S2: Reproducability table over HiSeqX vs. NovaSeq evaluation.

## Abbreviations

1KGP: 1000 Genomes Project; AWS: Amazon Web Services; bp: base pairs; CPU: central processing unit; GATK: Genome Analysis Toolkit; GIAB: Genome in a Bottle; kb: kilobase pairs; RAM: random access memory; SV: structural variant; WDL: Workflow Development Language; WGS: whole-genome sequence.

## Competing interests

This work was conducted when S.Z. and A.C. were employed at DNAnexus. Neither S.Z. nor A.C. is currently employed by DNAnexus and they do not have financial conflicts to disclose. F.J.S. has multiple sponsored travels from Oxford Nanopore and Pacific Biosciences and is the recipient of the 2018 SMRT Pacific Biosciences grant. The authors declare that they have no other competing interests.

## Funding

DNAnexus contributed computational resources and funding for personnel in this project. This work has been supported by NHGRI Centers for Common Disease Genomics (5UM1HG008898–02), ANVIL (5U24HG010263), and CCDG (UM1 HG008898). The funding body did not play any role in the design of the study and collection, analysis, and interpretation of data and in writing the manuscript.

## Authors' Contributions

S.Z. and A.C. implemented Parliament2 as a Docker image and a DNAnexus app. S.Z. implemented Parliament2 as a WDL workflow. F.J.S. implemented SURVIVOR and adapted it for Parliament2. S.Z., A.C., M.M., O.K., G.J., W.J.S., M.C.S., E.B., R.A.G., and F.J.S. contributed to writing the manuscript and study design.

## Supplementary Material

giaa145_GIGA-D-20-00101_Original_Submission

giaa145_GIGA-D-20-00101_Revision_1

giaa145_GIGA-D-20-00101_Revision_2

giaa145_Response_to_Reviewer_Comments_Original_Submission

giaa145_Response_to_Reviewer_Comments_Revision_1

giaa145_Reviewer_1_Report_Original_Submission Xuefang Zhao -- 4/23/2020 Reviewed

giaa145_Reviewer_1_Report_Revision_1 Xuefang Zhao -- 11/1/2020 Reviewed

giaa145_Reviewer_2_Report_Original_Submission Ryan Layer -- 5/9/2020 Reviewed

giaa145_Reviewer_2_Report_Revision_1 Ryan Layer -- 9/23/2020 Reviewed

giaa145_Supplemental_Files
