# Supplementary material for: Parliament2: Accurate structural variant calling at scale
Source: Gigascience. 2020 Dec 21;9(12):giaa145. doi: 10.1093/gigascience/giaa145 (PMC7751401; doi:10.1093/gigascience/giaa145)
Supplement: giaa145_GIGA-D-20-00101_Original_Submission [file giaa145_giga-d-20-00101_original_submission.pdf]

GigaScience

Parliament2: Accurate Structural Variant Calling At Scale

--Manuscript Draft--

**Manuscript Number:**	GIGA-D-20-00101
**Full Title:**	Parliament2: Accurate Structural Variant Calling At Scale
**Article Type:**	Technical Note
**Funding Information:**	National Institutes of Health (5UM1HG008898-02) — Dr Richard Gibbs
**Abstract:**	Background:<br>Structural variants (SVs) are critical contributors to genetic diversity and genomic disease. To predict the phenotypic impact of SVs, there is a need for better estimates of both the occurrence and frequency of SVs, preferably from large, ethnically diverse cohorts. Thus, the current standard approach requires the usage of short paired-end reads, which remain challenging to detect, especially at the scale of hundreds to thousands of samples.<br><br>Findings:<br>We present Parliament2, a consensus SV framework that leverages multiple best-in-class methods to identify high-quality SVs from short-read DNA sequence data at scale. Parliament2 incorporates pre-installed SV callers that are optimized for efficient execution in parallel processes, to reduce the overall runtime and costs. We demonstrate the accuracy of Parliament2 when applied to data from NovaSeq and HiSeq X platforms with the Genome in a Bottle SV call set across all size classes. The reported quality score per SV is inferred from the combination of supporting methods over different SV types and size classes. Parliament2 has the highest F1 score (74.27%) measured across the independent gold standard from GIAB. We illustrate the compute performance by processing all 1000 Genomes samples (2,691 samples) in less than a day on GRCH38. Parliament2 improves the runtime performance of individual methods, is open-source (https://github.com/dnanexus/parliament2) and a Docker image is available.<br><br>Conclusion:<br>Parliament2 provides both a highly accurate single-sample SV call set from short-read DNA sequence data and enables cost-efficient application over cloud or cluster environments, processing thousands of samples.
**Corresponding Author:**	Fritz Sedlazeck<br><br>UNITED STATES
**Corresponding Author Secondary Information:**	
**Corresponding Author's Institution:**	
**Corresponding Author's Secondary Institution:**	
**First Author:**	Samantha Zarate
**First Author Secondary Information:**	
**Order of Authors:**	Samantha Zarate<br>Andrew Carroll<br>Olga Krasheninina<br>Goo Jun<br>William Salerno<br>Eric Boerwinkle<br>Richard Gibbs<br>Fritz Sedlazeck

Powered by Editorial Manager® and ProduXion Manager® from Aries Systems Corporation

**Order of Authors Secondary Information:**	
**Additional Information:**	
**Question**	**Response**
Are you submitting this manuscript to a special series or article collection?	No
**Experimental design and statistics**<br><br>Full details of the experimental design and statistical methods used should be given in the Methods section, as detailed in our Minimum Standards Reporting Checklist. Information essential to interpreting the data presented should be made available in the figure legends.<br><br>Have you included all the information requested in your manuscript?	Yes
**Resources**<br><br>A description of all resources used, including antibodies, cell lines, animals and software tools, with enough information to allow them to be uniquely identified, should be included in the Methods section. Authors are strongly encouraged to cite Research Resource Identifiers (RRIDs) for antibodies, model organisms and tools, where possible.<br><br>Have you included the information requested as detailed in our Minimum Standards Reporting Checklist?	Yes
**Availability of data and materials**<br><br>All datasets and code on which the conclusions of the paper rely must be either included in your submission or deposited in publicly available repositories (where available and ethically appropriate), referencing such data using a unique identifier in the references and in the "Availability of Data and Materials" section of your manuscript.<br><br>Have you have met the above requirement as detailed in our Minimum Standards Reporting Checklist?	Yes

Powered by Editorial Manager® and ProduXion Manager® from Aries Systems Corporation

Manuscript

Click here to download Manuscript Manuscript.docx

Parliament2: Accurate Structural Variant Calling At Scale

Samantha Zarate[1,2], Andrew Carroll[1], Olga Krasheninina[3], Goo Jun[4], William J. Salerno[3], Eric Boerwinkle[3,4], Richard A. Gibbs[3], Fritz J Sedlazeck[3*]

1. DNAnexus, Mountain View, CA, 94040

2. Department of Computer Science, Johns Hopkins University, Baltimore, MD, USA

3. Human Genome Sequencing Center, Baylor College of Medicine, Houston TX 77030

4. Human Genetics Center, University of Texas Health Science Center at Houston, Houston TX, 77040

*Correspondence: fritz.sedlazeck@bcm.edu

Abstract

Background:

Structural variants (SVs) are critical contributors to genetic diversity and genomic disease. To predict the phenotypic impact of SVs, there is a need for better estimates of both the occurrence and frequency of SVs, preferably from large, ethnically diverse cohorts. Thus, the current standard approach requires the usage of short paired-end reads, which remain challenging to detect, especially at the scale of hundreds to thousands of samples.

Findings:

We present Parliament2, a consensus SV framework that leverages multiple best-in-class methods to identify high-quality SVs from short-read DNA sequence data at scale. Parliament2 incorporates pre-installed SV callers that are optimized for efficient execution in parallel processes, to reduce the overall runtime and costs. We demonstrate the accuracy of Parliament2 when applied to data from NovaSeq and HiSeq X platforms with the Genome in a Bottle SV call set across all size classes. The reported quality score per SV is inferred from the combination of supporting methods over different SV types and size classes. Parliament2 has the highest F1 score (74.27%) measured across the independent gold standard

from GIAB. We illustrate the compute performance by processing all 1000 Genomes samples (2,691 samples) in less than a day on GRCH38. Parliament2 improves the runtime performance of individual methods, is open-source (https://github.com/dnanexus/parliament2) and a Docker image is available.

Conclusion:

Parliament2 provides both a highly accurate single-sample SV call set from short-read DNA sequence data and enables cost-efficient application over cloud or cluster environments, processing thousands of samples.

Keywords: Structural Variation, Next Generation Sequencing, High throughput sequencing.

Findings

Structural variants (SVs) comprise a broad class of genomic alterations, typically defined as events 50 bp or larger, including deletions, duplications, insertions, inversions, and translocations. SVs are critical to fully understanding evolutionary processes, gene expression, and genomic diseases such as Mendelian disorders and cancer [1–3]. Accurate SV detection is limited by the inherent problem that SVs are generally larger than the short reads that compose the majority of sequencing data. Therefore, SVs are usually inferred by including split-read mapping, soft-clipped reads, changes in the distance between and orientation of read pairs, coverage depth variations, and alterations in the heterozygosity of a region [3,4]. Even best-in-class methods can fail to capture the majority of SVs (30% to 70% sensitivity) and often return a high false discovery rate, especially for insertion and inversion events [5,6].

Common SV detection methods, including Breakdancer [7], CNVnator [8], Crest [9], Delly [10], Lumpy [11], Manta [11,12], and Pindel [13], rely on heuristic approaches leveraging some or most of the mapped-read signals. This diversity of approaches also results in performance heterogeneity across SV types and size regimes as well as varied compute requirements. The differences in approaches also allow for ensemble optimization. Two methods, MetaSV [14] and Parliament [15], employ a three-step Overlap-Merge-Validate strategy to combine results of multiple callers into a high-quality consensus set. Both MetaSV and

Parliament use an assembly-based method for the validation step, which, while accurate, is computationally intensive and limits the maximum size of events [15]. Because MetaSV and Parliament start from existing SV calls, they place the burden of installing and running individual SV callers on the user. Furthermore, the computational requirements present additional challenges to at-scale execution for large sample sets.

Here we present Parliament2, a scalable SV caller optimized for cloud-based analysis with high precision and recall designed for single-sample analysis and large cohort aggregation. Parliament2 executes any combination of Breakdancer, Breakseq, CNVnator, Delly, Lumpy, and Manta to generate candidate SV events; uses SURVIVOR [16] to overlap these calls into consensus SVs candidates; validates these calls using SVTyper [16,17]; and for each event assigns a quality value derived from the SV size, type, and combination of supporting methods. Parliament2 reports multiple SV types including deletions, duplications, insertions, inversions, and translocations. Computational efficiency is achieved via multiple parallelization strategies that execute callers simultaneously, taking advantage of the complementary requirements in CPU, disk I/O, and RAM. This parallelization speeds up the individual methods and thus allows Parliament2 a faster execution time than running the programs on its own. A 16-core machine can process a 35x whole genome sequence (WGS) sample in two to five hours. Parliament2 is tunable in terms of recall and precision, meeting the needs of multiple experimental designs, such as maximal sensitivity in research settings and clinical-grade precision for diagnostics. Parliament2 has been tested across multiple platforms and optionally provides PDF images for manual curation using SVVIZ [18].

Parliament2 is open-source and available as a code base (https://github.com/dnanexus/parliament2), a DNAnexus app, and a Docker image that can be used to easily run any combination of individual callers (https://hub.docker.com/r/dnanexus/parliament2/).

Accuracy assessments for Parliament2 based on real data

We assessed the performance of Parliament2 in terms of precision (1 - False Discovery Rate), recall (True Positive Rate), and runtime compared to other short-read SV methods (using their default or otherwise suggested parameters) based on the Genome in a Bottle (GIAB) v0.6 SV candidate truth set [19] and using the suggested Truvari software (https://github.com/spiralgenetics/truvari) for comparing SV calls greater

than 50 bp. Parliament2 ran in 3.43 hours (wall time) on a 16-core machine from a 35x coverage BAM aligned to the hs37d5 reference genome. While Parliament2 can infer multiple SV types, the current GIAB call set largely comprises insertion and deletion events. Apart from other SV callers, we also benchmarked MetaSV, which also leverages multiple SV callers together. Due to the complexity of MetaSV, we used the results submitted by their authors to GIAB. **Figure 1a** shows the results for small deletions (50-300 bp) (see **Supplementary Table 1** for details). The vast majority of the GIAB call set includes 32,520 (86.92%) deletions of this size range highlighting its importance to detect these events. We obtained only deletion calls from Manta, Delly, and Breakseq for this size category. Parliament2 had the highest recall rate (56.54%) while having the third-highest precision (85.17%). Only Breakseq (93.20%) and Meta-SV (90.84%) had a higher precision, likely due to their having the lowest recall rates, calling only 15.69% and 18.04% of the deletions, respectively. Thus, Parliament2 (67.96%) had the highest F1 score, followed by Manta (64.00%). **Figure 1b** shows the performance of the different SV calling methods over the 3,278 (8.76%) mid-size deletions (300-1000 bp) (see **Supplementary Table 1** for details). Parliament2 had the second-highest recall (83.20%) and the highest precision (96.49%). Only MetaSV had a marginally higher recall (83.81%). Again, Parliament2 showed the highest F1 score (89.35%), followed by Manta (86.65%). For deletions larger than 1 kbp (**Figure 1c**; see **Supplementary Table 1** for details) only 1,614 (4.31%) of the gold standard, MetaSV showed the highest F1 score (89.83%) closely followed by Parliament2 (87.89%), both driven by their high precision scores of 91.92% and 91.59%, respectively. Across all size regimes for deletions, Parliament2 achieved by far the highest F1 score (average: 81.73%) followed by Manta (77.31%), MetaSV (68.06%), Delly (65.20%), Breakseq (63.28%), Breakdancer (58.78%), Lumpy (49.96%), and CNVnator (11.12%).

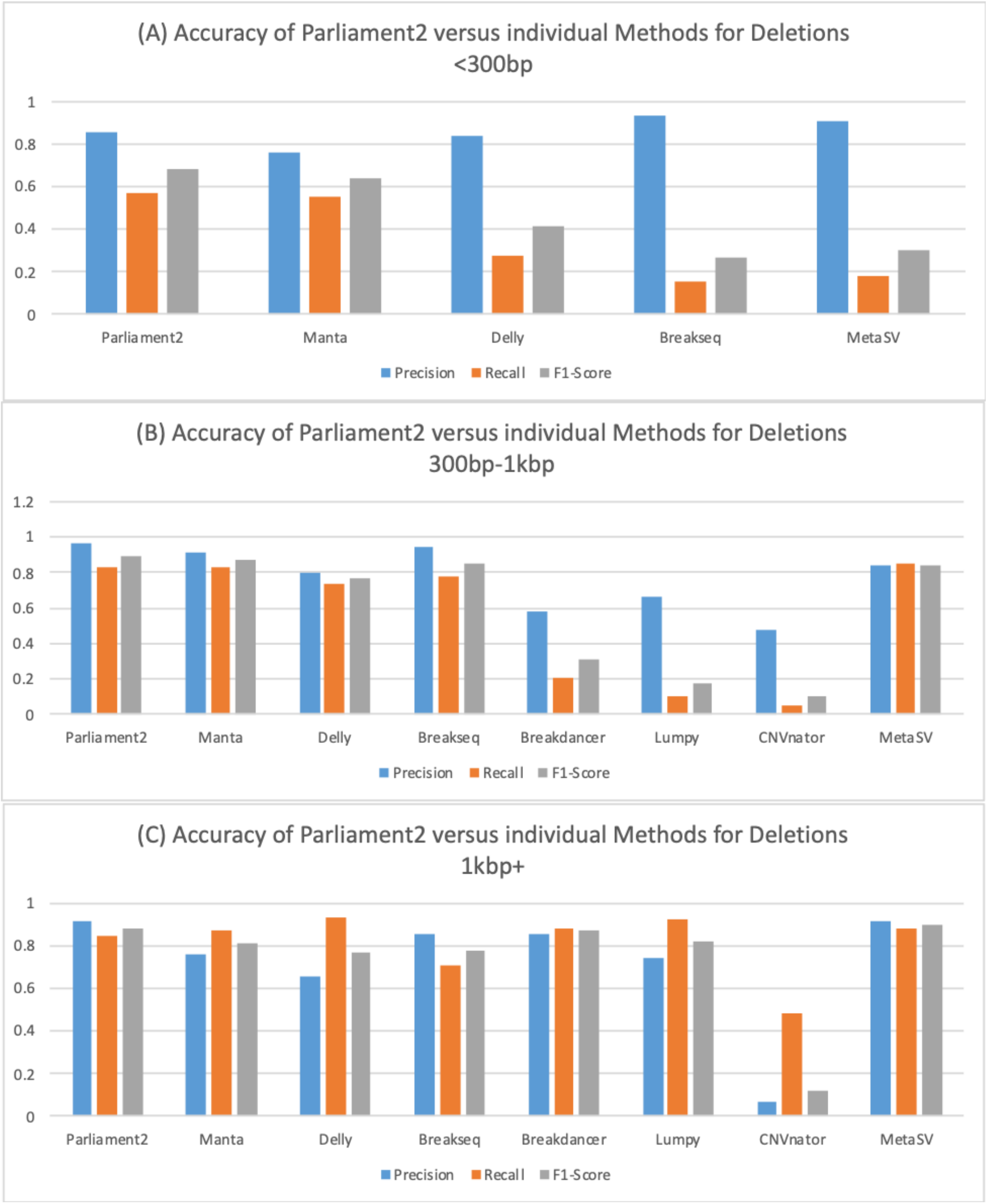

Figure 1. *Accuracy comparison for Parliament2 based on GIAB v0.6 deletion call set for different size regimes of deletions. (A) less than 300 bp, (B) between 300 bp and 1 kbp, and (C) larger than 1 kbp. The order of methods in each graph is sorted such that methods with higher F1 scores are located to the left. The efficacies of individual methods vary between size ranges.*

Compute Efficiency

Runtime and computational efficiency are essential to scalability and cost reduction. The SV callers used by Parliament2 fall into three parallelization classes: native multi-threading (Breakseq, Manta); native parallelization by chromosome (CNVnator, Breakdancer); and those lacking either (Delly, Lumpy). Upon execution, Parliament2 immediately executes Breakseq and Manta with multiple threads, splits the input BAM by chromosome, and initiates runs on the remaining callers. For the 35x HG002 BAM, this strategy reduced the runtime for Lumpy from 6.45 hours to 0.45 hours and for Delly from 8.52 hours to 0.67 hours on a 16-core machine.

The parallelization across multiple programs leads to a reduction in runtime by achieving higher overall machine utilization of resources (**Figure 2**). In local and cloud environments, this optimization translates to reductions of cost, CPU utilization, and wall time.

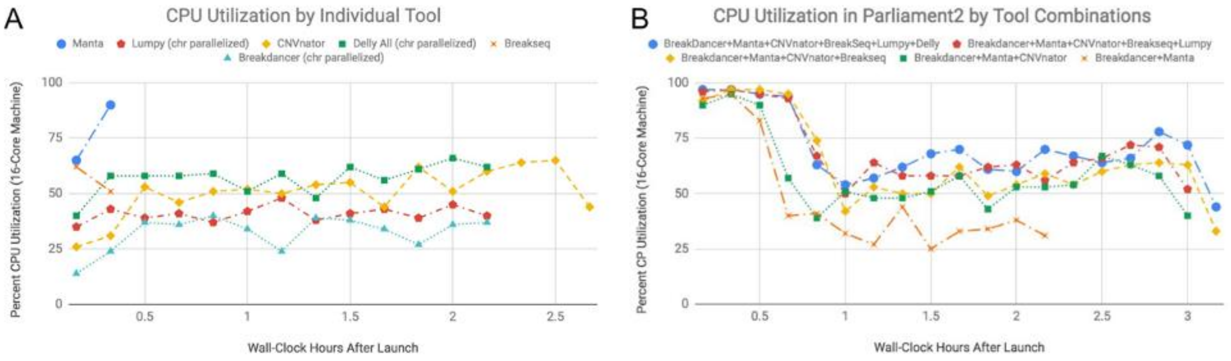

Figure 2. *Concurrent execution of multiple tools in Parliament2 increases resource utilization. (A) Percent of total CPU utilization on a 16-core machine executing Parliament2 and running only an individual tool. Each line terminates when the program finishes executing. (B) Resource utilization when running combinations of methods simultaneously within Parliament2.*

Consensus Quality Scores

One oft-discussed problem for short-read based SV calling is low sensitivity and high false discovery rates [5,6]. This challenge is exacerbated by the variety of SV types and sizes and the applicability of various methods to each SV class. The different performances of individual methods (see above) highlight the potential of a consensus approach stratified by size and event type. Without such a distinction, accuracy

assessments are dominated by the more numerous small events, potentially under-reporting rare but impactful gene-sized events.

We analyzed the contribution of each Parliament2 caller to the overall precision. **Figure 3a** describes how each combination of SV methods contributes to recall performance. The precision of SV calls obtained by a single individual method ranges from 8% with CNVnator to 91% for Breakseq. However, when an SV call is supported by multiple methods, precision can reach 100% independent of the size regime (**Figure 3a, 3b**). The combination of CNVnator and BreakSeq is the minimum set of SV callers to reach 100% precision. Although CNVnator has the lowest precision performance (8%), it is included in every set that reaches a 100% recall rate. Thus, while deletions discovered only by CNVnator have low precision, a deletion call from CNVnator and at least one other method provides high precision. While only a few methods (Breakseq, Manta, and Delly) detect insertions, they are generally precise (98%-100%). **Supplementary Figure 1** shows the precision of the individual SV caller and their combinations for insertions.

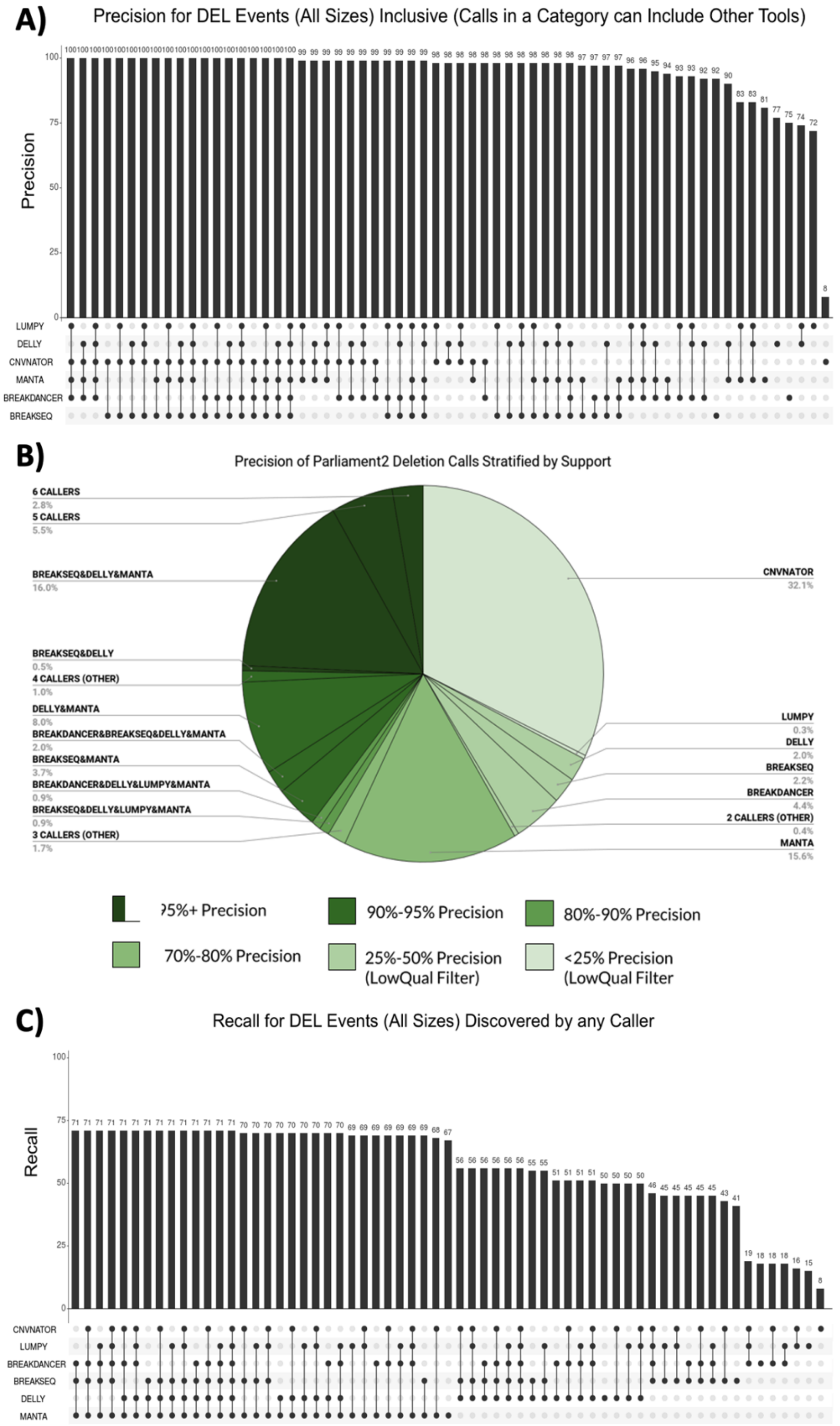

Figure 3: *Assessment of constituent SV calling methods based on the deletion call set from GIAB v0.6. A) Measured precision for the different method combinations. The precision ranges from 5% (CNVnator) up to 100% for various combinations. B) Contributions of the individual SV callers and their combinations to the total number of calls (percent label) and their relative*

precision (color-coded by shade of green). C) Measured recall for individual methods and their combinations ranging from 8% (CNVnator) to 71% for various combinations.

Figure 3c details the recall rates of individual SV callers and their combinations. The highest recall rate (71%) is achieved by a combination of multiple callers. This value is surprisingly high given that the truth set includes data from multiple long-read technologies and SVs that were only obtained by long-read sequencing and assembly. Manta is included in all of the combinations that reached a high recall value for deletions. For insertions, the overall recall is drastically reduced to 17% using a combination of Manta (15%), Delly (3%), and BreakSeq (2%) (**Supplementary Figure 1**)

Based on these observations, we generated a ruleset based on GIAB deletion calls assuming the individual SV callers show similar metrics in other types of SVs. This ruleset is then applied to assign quality values to each of the reported SV calls. Parliament2 expresses the call quality as a Phred-encoded value within the final consensus VCF. These scores are based on the precision results from GIAB for each combination of supporting callers, the type of the event (deletion or insertion), and the size category of the event (50-300 bp, 300-1000 bp, >1 kbp). This quality value allows investigators to set thresholds to achieve the trade-off between precision and recall that is desired for their use cases or to prioritize events based on how likely they are to be true events. **Supplementary Figure 1** shows the quality value based on caller support and the SVs type and size. These quality values enable Parliament2 to obtain a balanced performance for recall rate and precision resulting in the highest F1 scores (**Figure 1a-c**) across multiple size regimes. The same ruleset is also applied to other SV types for which we lacked GIAB benchmark data (e.g. inversions).

Inter-Platform Concordance

Large collaborative projects aggregate heterogeneous data across different sequencing centers, chemistry versions, and short-read platforms (e.g. HiSeq X and NovaSeq). Given the inferential nature of SV detection from short reads, SV methods are particularly susceptible to batch effects. Therefore, we have characterized Parliament2 using HiSeq X and NovaSeq sequencing runs, including the HiSeq X data described above and four NovaSeq HG002 replicates each downsampled to 35x coverage and mapped to the hs37d5 reference. These 35x NovaSeq replicates showed similar precision (83.0%) and recall (69.35%)

compared to the HiSeq X (81.7% and 70.7%, respectively). Increasing coverage to 50x for all samples across both platforms changed these values by <5% (see **Supplementary Table 2** for details), indicating the robustness of evaluating both platforms at 35x. The unfiltered concordance values, corresponding to all raw Parliament2 consensus calls, indicate low inter-platform consistency, which would likely drive batch effects in mixed-platform sample sets. After filtering for Parliament2 events with a quality value greater than 3, inter- and intra-platform concordances increase to similar levels, suggesting both an increase in quality and mitigation of platform batch effects.

1000 Genomes Project SVs for GRCh38

The 1000 Genomes Project (1KGP) is a valuable resource of high-confidence SV calls across a large sample set (2,691 samples) mapped to GRCh37. However, since the introduction of GRCh38 [20], many large-scale whole-genome programs (e.g. TOPMed, All of Us) have adopted this standard. To demonstrate the scalability of Parliament2 for large datasets and to create a community resource, we applied Parliament2 to the 2,691 1KGP WGS samples mapped to GRCh38 [20]. Although the 1KGP samples have been remapped to GRCh38 [20,21], we are not aware of a comprehensive set of SVs on these data and reference sets.

The computational requirements were modest in comparison to other familiar applications, and the entire SV calling was completed in one day of wall clock time, using only 63,720 CPU-hours (on average 24 core-hours per sample). For reference, that amount of compute is approximately equivalent to running GATK4 on 220 WGS samples at 35x coverage. This effort created SVs calls for each of Breakdancer, CNVnator, Delly, Lumpy, and Manta, as well as SVTyped files of each and consensus Parliament2 calls. **Figure 5** shows the results for these call sets.

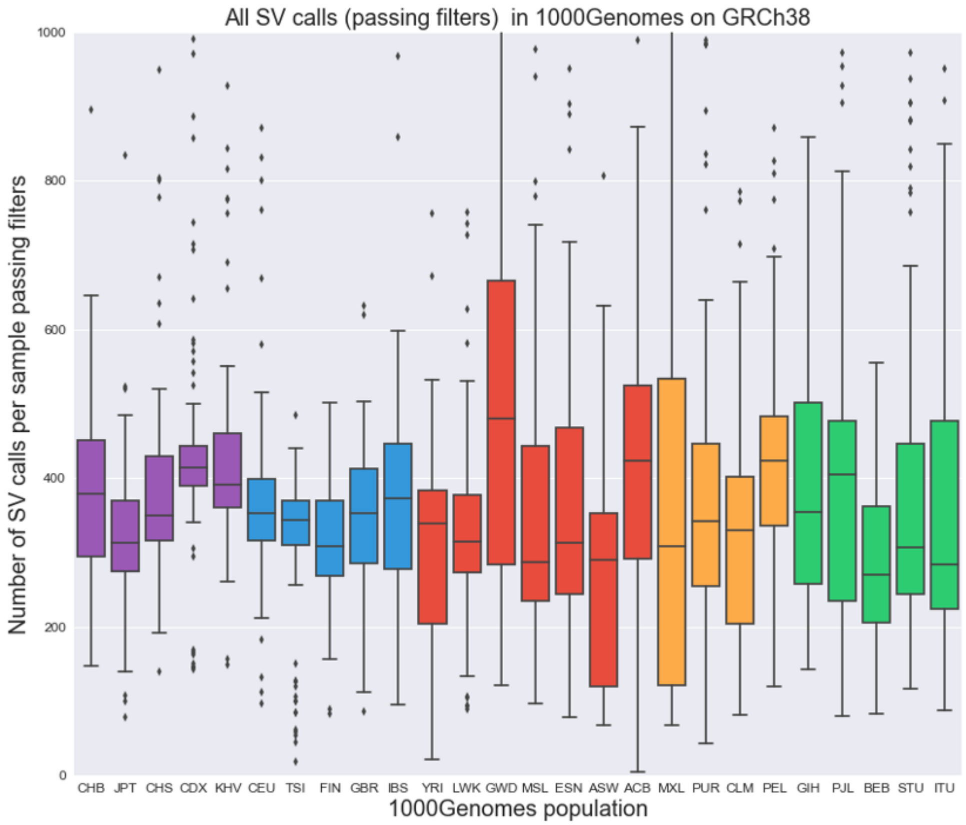

Figure 5. *Population distribution of SV calls produced by Parliament2 for the 1000Genomes Phase3.*

Figure 5 shows the distribution of all SV calls that pass quality filters per sample in the 1000genomes. Populations are colored by their super population code: EAS-purple, EUR-blue, AFR-red, AMR-orange, SAS-green.

In total, there are 88,404 deletions larger than 50 bp discovered in this set and 30,479 inversion events. There were only 619 insertions discovered, possibly reflecting that it is more difficult to detect an insertion in these low coverage data. The number of calls per sample was generally lower than observed for the high-coverage WGS samples investigated in the prior benchmarks. Additionally, for certain samples, some of the callers did not generate any output, possibly due to low sequence coverage of the samples. These SV calls will provide a resource to understand SVs called on GRCh38 relative to the multiple ethnicities captured in 1KGP and to understand how each of these tools interacts with lower coverage data.

The Parliament2 optimized consensus approach addresses the accuracy and compute challenges of calling SVs from short-read sequence data at scale. Leveraging consensus calling for event discovery and quality assessment, Parliament2 achieves a higher overall accuracy (F1 score against GIAB HG002 SVs) than any constituent method without compromising efficiency, providing robust SV calling across multiple platforms. Parliament2 is unique in its capability to identify multiple classes of SVs in an easily scaled manner, enabling efficient computation on a single sample (~3 hours) or on thousands of samples. Within one day of Parliament2 compute, we have generated the first comprehensive SV set for the 1KGP samples on GRCh38, a publicly available resource (see **Supplement**).

Parliament2 specializes relative to MetaSV in two key ways. First, Parliament2 is optimized for scalability, not requiring an expert user to launch multiple SV callers, the results of which need to be combined later (e.g. SURVIVOR, MetaSV). This leads to a faster and more efficient execution over thousands of samples. Parliament2 pre-installs these programs, configures them to speed up the processing, and utilizes a trained quality value to provide extra information about the reliability of the SV calls. Second, MetaSV does not provide a full workflow and includes costly assembly steps that result in high computational costs over multiple samples. Nevertheless, these enable MetaSV to report sequence-resolved insertion calls, while Parliament2 can only produce the sequence resolution if the individual method that called the event produced it. Still, this complicates the execution of MetaSV over multiple hundred to thousands of samples required for larger cohorts.

SV calling accuracy, however, remains an open challenge. F1 scores for best-in-class small variant callers routinely exceed 99%, and even higher standards are required for clinical reporting. As SV methods improve, the Parliament2 infrastructure can be easily adapted to incorporate new methods (e.g. graph-based references and rapid local assembly) and SV callers, especially those that target specific SV types such as mobile element insertions and variable nucleotide tandem repeats, to determine the optimal consensus strategy. Such improvement will be accelerated by broader and deeper high-confidence SVs from long-range data across more samples and ethnicities against which SV methods such as Parliament2 can be trained.

Methods

Parliament2 Implementation

The code for Parliament2 is in a GitHub repository with an open-source (Apache-2.0) license at the 1.0.7 version (commit 97517b1a22104a3e0a0966a79c3b5556fde8a89d). Execution of Parliament2 done by running v1.0.7 of the Parliament2 DNAnexus app (app-FJ8Fj88054JxXFygKvFqQ39j), which is publicly available to run by any user on DNAnexus. This app runs a Docker image built directly from the GitHub repository. Executions of the app with user-provided input for tool combinations specify the parameter flags to the Docker image to include or exclude the desired tools.

Input WGS Data Used for Timing and Accuracy Benchmarks

Timing statistics and resource utilization were determined by executing the Parliament2 app on a 35X WGS sample for HG002 that was made by random downsampling the 50X PCR-Free HG002 HiSeqX sample generated for the Challenge set of the PrecisionFDA Truth Challenge.

Timing for individual tools and Parliament2 combinations

All timing calculations are run on a c3.4xlarge AWS instance (16-core, 30GB RAM, 320GB disk). To calculate the runtime and resource utilization of individual components, the Parliament2 app was launched with the desired tool or tool combinations. DNAnexus apps write an entry of machine resources (CPU percent, RAM, and disk utilization) every 10 minutes to a job log that also contains the stdout and stderr outputs for job execution. All info log entries of this after the stderr line for program execution up until the SVTyper step (which indicates completion of all jobs) were taken to determine the resource plots over time.

The logs for these jobs are available at:
https://github.com/dnanexus/parliament2/tree/master/benchmarking_data/dx_job_logs

Accuracy comparisons

Accuracy comparisons are performed using Truvari (https://github.com/spiralgenetics/truvari) with the following execution: truvari.py -b GIAB_DEL0.6.vcf.gz -c <parliament_output> -o <output_directory --passonly --includebed GIAB_0.6.bed --pctsim=0 -r 2000 –giabreport

To determine accuracy for size ranges, -s <lower_size> and -S <upper_size> were used. The deletion truth set was taken by extracting SVTYPE=DEL from the v0.6 truth set. The insertion truth set was taken similarly by extracting SVTYPE=INS.

The Genome in a Bottle data for v0.6 truth set is at:

ftp://ftp-trace.ncbi.nlm.nih.gov/giab/ftp/data/AshkenazimTrio/analysis/NIST_SVs_Integration_v0.6/

Tool Versions

The individual tools that comprise Parliament2 run the following versions of each program:

Breakdancer: [v1.4.3]

(https://github.com/genome/breakdancer/releases/tag/v1.4.3)

BreakSeq2:[v2.2]

(http://bioinform.github.io/breakseq2/)

CNVnator:[v0.3.3]

(https://github.com/abyzovlab/CNVnator/commit/de012f2bccfd4e11e84cf685b19fc138115f2d0d)

Delly:[v0.7.2]

(https://github.com/dellytools/delly/releases/tag/v0.7.2)

Lumpy: [v0.2.13]

(https://github.com/arq5x/lumpy-sv/commit/f466f61e02680796192b055e4c084fbb23dcc692)

Manta: [v1.4.0]

(https://anaconda.org/bioconda/manta)

Availability of supporting source code and requirements

Project name: Parliament2

Project home page: https://github.com/dnanexus/parliament2

Operating system(s): Linux

Programming language: Python/C++

Other requirements:

Availability of supporting data

Benchmark output:

All benchmark results of all the programs can be found:

https://github.com/dnanexus/parliament2/tree/master/benchmarking_data/hg002_benchmarks

1000 genome download links for the following resources are:

A project-level VCF of all PASS and unfiltered variants in any sample:

https://github.com/dnanexus/parliament2/tree/master/benchmarking_data/1000_genomes

The VCF output of Parliament2:

https://github.com/dnanexus/parliament2/tree/master/benchmarking_data/hg002_benchmarks

The individual caller files for Breakdancer, BreakseqCNVnator, Delly, Lumpy, and Manta are available at:

https://github.com/dnanexus/parliament2/tree/master/benchmarking_data/hg002_benchmarks/sv_caller_outputs

Competing interests

This work was conducted when SZ and AC were employed at DNAnexus. Neither SZ nor AC are currently employed by DNAnexus and do not have financial conflicts to disclose. FJS has multiple sponsored travels from Oxford Nanopore and PacBio and is the recipient of the 2018 SMRT PacBio grant.

Funding

DNAnexus contributed computational resources and funding for personnel in this project. This work has been supported by NHGRI Centers for Common Disease Genomics grant 5UM1HG008898-02). The funding body did not play any roles in the design of the study and collection, analysis, and interpretation of data and in writing the manuscript.

Authors' contributions

SZ and AC implemented Parliament2 as a Docker image and a DNAnexus app. FJS implemented SURVIVOR and adopted it for Parliament2. SZ,AC,OK,GJ,WJS,EB,RAG, and FJS contributed in writing the manuscript and study design.

Acknowledgements

References

1. Feuk L, Carson AR, Scherer SW. Structural variation in the human genome [Internet]. Nature Reviews Genetics. 2006. p. 85–97. Available from: http://dx.doi.org/10.1038/nrg1767

2. Lupski JR. Structural variation mutagenesis of the human genome: Impact on disease and evolution. Environ Mol Mutagen. 2015;56:419–36.

3. Mahmoud M, Gobet N, Cruz-Dávalos DI, Mounier N, Dessimoz C, Sedlazeck FJ. Structural variant calling: the long and the short of it. Genome Biol. 2019;20:246.

4. Alkan C, Coe BP, Eichler EE. Genome structural variation discovery and genotyping [Internet]. Nature Reviews Genetics. 2011. p. 363–76. Available from: http://dx.doi.org/10.1038/nrg2958

5. Sedlazeck FJ, Rescheneder P, Smolka M, Fang H, Nattestad M, von Haeseler A, et al. Accurate detection of complex structural variations using single-molecule sequencing. Nat Methods. 2018;15:461–8.

6. Chaisson MJP, Huddleston J, Dennis MY, Sudmant PH, Malig M, Hormozdiari F, et al. Resolving the complexity of the human genome using single-molecule sequencing. Nature. 2015;517:608–11.

7. Chen K, Wallis JW, McLellan MD, Larson DE, Kalicki JM, Pohl CS, et al. BreakDancer: an algorithm for high-resolution mapping of genomic structural variation. Nat Methods. 2009;6:677–81.

8. Abyzov A, Urban AE, Snyder M, Gerstein M. CNVnator: an approach to discover, genotype, and characterize typical and atypical CNVs from family and population genome sequencing. Genome Res. 2011;21:974–84.

9. Wang J, Mullighan CG, Easton J, Roberts S, Heatley SL, Ma J, et al. CREST maps somatic structural variation in cancer genomes with base-pair resolution. Nat Methods. 2011;8:652–4.

10. Rausch T, Zichner T, Schlattl A, Stütz AM, Benes V, Korbel JO. DELLY: structural variant discovery by integrated paired-end and split-read analysis. Bioinformatics. 2012;28:i333–9.

11. Layer RM, Chiang C, Quinlan AR, Hall IM. LUMPY: a probabilistic framework for structural variant discovery. Genome Biol. 2014;15:R84.

12. Chen X, Schulz-Trieglaff O, Shaw R, Barnes B, Schlesinger F, Källberg M, et al. Manta: rapid detection of structural variants and indels for germline and cancer sequencing applications. Bioinformatics. 2016;32:1220–2.

13. Ye K, Schulz MH, Long Q, Apweiler R, Ning Z. Pindel: a pattern growth approach to detect break points of large deletions and medium sized insertions from paired-end short reads [Internet]. Bioinformatics. 2009. p. 2865–71. Available from: http://dx.doi.org/10.1093/bioinformatics/btp394

14. Mohiyuddin M, Mu JC, Li J, Bani Asadi N, Gerstein MB, Abyzov A, et al. MetaSV: an accurate and integrative structural-variant caller for next generation sequencing. Bioinformatics. 2015;31:2741–4.

15. English AC, Salerno WJ, Hampton OA, Gonzaga-Jauregui C, Ambreth S, Ritter DI, et al. Assessing structural variation in a personal genome—towards a human reference diploid genome [Internet]. BMC Genomics. 2015. Available from: http://dx.doi.org/10.1186/s12864-015-1479-3

16. Jeffares DC, Jolly C, Hoti M, Speed D, Shaw L, Rallis C, et al. Transient structural variations have strong effects on quantitative traits and reproductive isolation in fission yeast [Internet]. Nature Communications. 2017. Available from: http://dx.doi.org/10.1038/ncomms14061

17. Chiang C, Layer RM, Faust GG, Lindberg MR, Rose DB, Garrison EP, et al. SpeedSeq: ultra-fast personal genome analysis and interpretation. Nat Methods. 2015;12:966–8.

18. Spies N, Zook JM, Salit M, Sidow A. svviz: a read viewer for validating structural variants. Bioinformatics. 2015;31:3994–6.

19. Zook JM, Hansen NF, Olson ND, Chapman LM, Mullikin JC, Xiao C, et al. A robust benchmark for germline structural variant detection [Internet]. Available from: http://dx.doi.org/10.1101/664623

20. Sudmant PH, Rausch T, Gardner EJ, Handsaker RE, Abyzov A, Huddleston J, et al. An integrated map of structural variation in 2,504 human genomes. Nature. 2015;526:75–81.

21. Zheng-Bradley X, Streeter I, Fairley S, Richardson D, Clarke L, Flicek P, et al. Alignment of 1000 Genomes Project reads to reference assembly GRCh38. Gigascience. 2017;6:1–8.

Supplement Tables

Click here to access/download
Supplementary Material
Parliament2_tables.xlsx

supplement figures

Click here to access/download
Supplementary Material
Figures_Parliament2.pptx

Coverletter
Click here to download Personal Cover Cover_Letter.docx

Baylor College of Medicine
Human Genome Sequencing Center

One Baylor Plaza
Houston, Texas 77030-3498

TEL: (713)798-6539
FAX: (713)798-5741

April 3rd, 2020

Dear Editor,
Please find enclosed a copy of our manuscript titled "Parliament2: Fast Structural Variant Calling Using Optimized Combinations of Callers", which we submit for consideration to *Gigascience*.

In our manuscript, we address the challenge of detecting structural variants using short-read data at precision and scale. Parliament2 enables users to accurately call SVs for large-scale populations, leveraging best-in-class SV callers and executing them optimally.
As you know, small-variant (<20bp) methods can achieve 99% and higher levels of accuracy. However, the sensitivity and specificity of structural variant detection are both widely agreed to be far lower. Furthermore, most of the current methods are not conducive to a large scale analysis utilizing cloud infrastructure. Thus, Parliament2 was designed to overcome these challenges. Here we use the newly adopted Genome in a Bottle (GIAB) structural variant truth set to quantify the limitations of existing methods. Parliament2 combines the SV calls from these methods and thus outperforms them. We show that the overlap of methods allows many calls to be identified with very high precision (99% and higher) and with a boost in accuracy (sensitivity and precision). Furthermore, we use the GIAB truth set to calibrate confidence in each call by the overlap of supporting methods. This is the first time an independent measure of accuracy has enabled reliable quality scores for short-read structural variant calls.

To ensure broad adoption, we have optimized each method to run in parallel on a single machine and packaged a Docker image with all callers pre-installed. Even if a scientist only wants to run one of the methods, they can do so faster and without difficult installation requirements by running Parliament2. We believe this will contribute to its use and citation in the community.

Finally, we prove the scalability of Parliament2 by generating structural variant calls for the new hg38 mappings from the 2,691 1000 Genomes Phase 3 samples. We believe that this represents the first structural variant callset produced for the hg38 reference on this important dataset. Parliament2 accomplished this within a day of computation.

Thus, we are confident that this work will have a high degree of impact and citations and is also of interest to the readers of *Gigascience*. The calls generated by Parliament2 are already being used in several large whole-genome sequencing cohorts (CCDG, TopMed, etc) containing over 220K human samples. Independent researchers have already deployed Parliament2 in the NIH Biowulf cluster, Seven Bridges Genomics and on the NCI-funded Firecloud, in addition to its deployment on DNAnexus.

Consequently, we believe this work will lead to a better understanding of the genome by allowing accurate structural variant discovery at scale in both the human population and those of other species.

Best regards,

Fritz J. Sedlazeck, Ph.D.

Assistant Professor,
Human Genome Sequencing Center,
Baylor College of Medicine,
Houston, TX, 77030
