# Supplementary material for: Parliament2: Accurate structural variant calling at scale
Source: Gigascience. 2020 Dec 21;9(12):giaa145. doi: 10.1093/gigascience/giaa145 (PMC7751401; doi:10.1093/gigascience/giaa145)
Supplement: giaa145_GIGA-D-20-00101_Revision_1 [file giaa145_giga-d-20-00101_revision_1.pdf]

GigaScience

Parliament2: Accurate Structural Variant Calling At Scale

--Manuscript Draft--

**Manuscript Number:**	GIGA-D-20-00101R1
**Full Title:**	Parliament2: Accurate Structural Variant Calling At Scale
**Article Type:**	Technical Note
**Funding Information:**	National Institutes of Health (5UM1HG008898-02) — Dr Richard Gibbs<br>National Human Genome Research Institute (5U24HG010263) — Dr. Michael C Schatz
**Abstract:**	Background:<br>Structural variants (SVs) are critical contributors to genetic diversity and genomic disease. To predict the phenotypic impact of SVs, there is a need for better estimates of both the occurrence and frequency of SVs, preferably from large, ethnically diverse cohorts. Thus, the current standard approach requires the usage of short paired-end reads, which remain challenging to detect, especially at the scale of hundreds to thousands of samples.<br>Findings:<br>We present Parliament2, a consensus SV framework that leverages multiple best-in-class methods to identify high-quality SVs from short-read DNA sequence data at scale. Parliament2 incorporates pre-installed SV callers that are optimized for efficient execution in parallel to reduce the overall runtime and costs. We demonstrate the accuracy of Parliament2 when applied to data from NovaSeq and HiSeq X platforms with the Genome in a Bottle (GIAB) SV call set across all size classes. The reported quality score per SV is calibrated across different SV types and size classes. Parliament2 has the highest F1 score (74.27%) measured across the independent gold standard from GIAB. We illustrate the compute performance by processing all 1000 Genomes samples (2,691 samples) in less than a day on GRCH38. Parliament2 improves the runtime performance of individual methods, is open-source (https://github.com/dnanexus/parliament2), and a Docker image as well as a WDL implementation are available.<br>Conclusion:<br>Parliament2 provides both a highly accurate single-sample SV call set from short-read DNA sequence data and enables cost-efficient application over cloud or cluster environments, processing thousands of samples.
**Corresponding Author:**	Fritz Sedlazeck<br><br>UNITED STATES
**Corresponding Author Secondary Information:**	
**Corresponding Author's Institution:**	
**Corresponding Author's Secondary Institution:**	
**First Author:**	Samantha Zarate
**First Author Secondary Information:**	
**Order of Authors:**	Samantha Zarate<br>Andrew Carroll<br>Medhat Mahmoud<br>Olga Krasheninina<br>Goo Jun<br>William Salerno

Powered by Editorial Manager® and ProduXion Manager® from Aries Systems Corporation

	Michael C Schatz
	Eric Boerwinkle
	Richard Gibbs
	Fritz Sedlazeck
**Order of Authors Secondary Information:**	
**Response to Reviewers:**	We have attached the responses as the cover letter in the submission. The response also includes two new figures that would be lost in here.
**Additional Information:**	
**Question**	**Response**
Are you submitting this manuscript to a special series or article collection?	No
**Experimental design and statistics**<br><br>Full details of the experimental design and statistical methods used should be given in the Methods section, as detailed in our Minimum Standards Reporting Checklist. Information essential to interpreting the data presented should be made available in the figure legends.<br><br>Have you included all the information requested in your manuscript?	Yes
**Resources**<br><br>A description of all resources used, including antibodies, cell lines, animals and software tools, with enough information to allow them to be uniquely identified, should be included in the Methods section. Authors are strongly encouraged to cite Research Resource Identifiers (RRIDs) for antibodies, model organisms and tools, where possible.<br><br>Have you included the information requested as detailed in our Minimum Standards Reporting Checklist?	Yes
**Availability of data and materials**<br><br>All datasets and code on which the	Yes

conclusions of the paper rely must be either included in your submission or deposited in publicly available repositories (where available and ethically appropriate), referencing such data using a unique identifier in the references and in the "Availability of Data and Materials" section of your manuscript.<br><br>Have you have met the above requirement as detailed in our Minimum Standards Reporting Checklist?	

Powered by Editorial Manager® and ProduXion Manager® from Aries Systems Corporation

Manuscript Click here to access/download;Manuscript;Manuscript.docx

Parliament2: Accurate Structural Variant Calling At Scale

Samantha Zarate[1,2], Andrew Carroll[1], Medhat Mahmoud[3], Olga Krasheninina[3], Goo Jun[5], William J. Salerno[3], Michael C. Schatz[2], Eric Boerwinkle[3,4], Richard A. Gibbs[3], Fritz J Sedlazeck[3*]
1. DNAnexus, Mountain View, CA 94040
2. Department of Computer Science, Johns Hopkins University, Baltimore, MD 21218
3. Human Genome Sequencing Center, Baylor College of Medicine, Houston, TX 77030
4. Human Genetics Center, University of Texas Health Science Center at Houston, Houston, TX 77040

SZ: slzarate@jhu.edu
AC: acarroll.dna@gmail.com
MM: medhat.mahmoud@bcm.edu
OK: olga.krasheninina@regeneron.com
FJS: fritz.sedlazeck@bcm.edu
GJ: Goo.Jun@uth.tmc.edu
WS: william.salerno@regeneron.com
EB: Eric.Boerwinkle@uth.tmc.edu
RG: agibbs@bcm.edu

*Correspondence: fritz.sedlazeck@bcm.edu

Abstract

Background:
Structural variants (SVs) are critical contributors to genetic diversity and genomic disease. To predict the phenotypic impact of SVs, there is a need for better estimates of both the occurrence and frequency of SVs, preferably from large, ethnically diverse cohorts. Thus, the current standard approach requires the usage of short paired-end reads, which remain challenging to detect, especially at the scale of hundreds to thousands of samples.

Findings:
We present Parliament2, a consensus SV framework that leverages multiple best-in-class methods to identify high-quality SVs from short-read DNA sequence data at scale. Parliament2 incorporates pre-installed SV callers that are optimized for efficient execution in parallel to reduce the overall runtime and costs. We demonstrate the accuracy of Parliament2 when applied to data from NovaSeq and HiSeq X platforms with the Genome in a Bottle (GIAB) SV call set across all size classes. The reported quality score per SV is calibrated across different SV types and size classes. Parliament2 has the highest F1 score (74.27%) measured across the independent gold standard from GIAB. We illustrate the compute performance by processing all 1000 Genomes samples (2,691 samples) in less than a day on GRCH38. Parliament2 improves the runtime performance of individual methods, is open-source (https://github.com/dnanexus/parliament2), and a Docker image as well as a WDL implementation are available.

Conclusion:
Parliament2 provides both a highly accurate single-sample SV call set from short-read DNA sequence data and enables cost-efficient application over cloud or cluster environments, processing thousands of samples.

Keywords: Structural Variation, Next Generation Sequencing, High throughput sequencing.

Findings

Structural variants (SVs) comprise a broad class of genomic alterations, typically defined as events 50 bp or larger, including deletions, duplications, insertions, inversions, and translocations. SVs are critical to fully understanding evolutionary processes, gene expression, and genomic diseases such as Mendelian disorders and cancer [1–3]. Accurate SV detection is limited by the inherent problem that SVs are generally larger than the short reads that compose the majority of sequencing data. Therefore, SVs are usually inferred by including split-read mapping, soft-clipped reads, changes in the distance between and orientation of read-pairs, coverage depth variations, and alterations in the heterozygosity of a region [3,4]. Even best-in-class methods can fail to capture the majority of SVs (30% to 70% sensitivity) and often return a high false discovery rate, especially for insertion and inversion events [5,6].

Commonly used SV detection methods, such as Breakdancer [7], CNVnator [8], Crest [9], Delly [10], Lumpy [11], Manta [11,12], and Pindel [13], rely on heuristic approaches leveraging some or most of the mapped-read signals. This diversity of approaches also results in performance heterogeneity across SV types and size regimes as well as varied compute requirements. The differences in approaches also allow for ensemble optimization. Two methods, MetaSV [14] and Parliament [15] employ a three-step Overlap-Merge-Validate strategy to combine results of multiple callers into a high-quality consensus set. Both MetaSV and Parliament use an assembly-based method for the validation step, which, while accurate, is computationally intensive and limits the maximum size of events [15]. Because MetaSV and Parliament start from existing SV calls, they place the burden of installing and running individual SV callers on the user. Furthermore, the computational requirements present additional challenges to at-scale execution for large sample sets.

Here we present Parliament2, a scalable SV caller optimized for cloud-based analysis with high precision and recall designed for single-sample analysis and large cohort aggregation. Parliament2 executes any combination of Breakdancer, Breakseq, CNVnator, Delly, Lumpy, and Manta to generate candidate SV events; uses SURVIVOR [16] to overlap these calls into consensus SVs candidates; validates these calls using SVTyper [16,17]; and for each event assigns a quality value derived from the SV size, type, and combination of supporting methods. Parliament2 reports multiple SV types including deletions, duplications, insertions, inversions, and translocations. Computational efficiency is achieved via multiple parallelization strategies that execute callers simultaneously, taking advantage of the complementary requirements in CPU, disk I/O, and RAM. This parallelization speeds up the individual methods and thus allows Parliament2 a faster execution time than running the programs on its own. A 16-core machine can process a 35x whole genome sequence (WGS) sample in two to five hours. Parliament2 is tunable in terms of recall and precision, meeting the needs of multiple experimental designs, such as maximal sensitivity in research settings and clinical-grade precision for diagnostics. Parliament2 has been tested across multiple platforms and optionally provides PDF images for manual curation using SVVIZ [18].

Parliament2 is open-source and available as a code base (https://github.com/dnanexus/parliament2), a DNAnexus app, and a Docker image that can be used to easily run any combination of individual callers (https://hub.docker.com/r/dnanexus/parliament2/).

Accuracy assessments for Parliament2 based on real data

We assessed the performance of Parliament2 in terms of precision (1 - False Discovery Rate), recall (True Positive Rate), and runtime compared to other short-read SV methods (using their default or otherwise

suggested parameters) based on the Genome in a Bottle (GIAB) v0.6 SV candidate truth set [19] and using the suggested Truvari software (https://github.com/spiralgenetics/truvari) for comparing SV calls greater than 50 bp. The GIAB SV truth set is based on HG002, a male Ashkenazi Jewish sample utilizing multiple technologies and manual vetting of the SV. Parliament2 ran in 3.43 hours (wall time) for this sample on a 16-core machine from a 35x coverage BAM aligned to the hs37d5 reference genome. While Parliament2 can infer multiple SV types, the current GIAB call set largely comprises insertion and deletion events. Apart from other SV callers, we also benchmarked MetaSV, which also leverages multiple SV callers together. Due to the complexity of MetaSV, we used the results submitted by their authors to GIAB. **Figure 1a** shows the results for small deletions (50-300 bp) (see **Supplementary Table 1** for details). The vast majority of the GIAB call set includes 32,520 (86.92%) deletions of this size range highlighting its importance to detect these events. We obtained only deletion calls from Manta, Delly, and Breakseq for this size category. Parliament2 had the highest recall rate (56.54%) while having the third-highest precision (85.17%). Only Breakseq (93.20%) and Meta-SV (90.84%) had a higher precision, likely due to their having the lowest recall rates, calling only 15.69% and 18.04% of the deletions, respectively. Thus, Parliament2 (67.96%) had the highest F1 score (i.e. harmonic mean of precision and recall), followed by Manta (64.00%). **Figure 1b** shows the performance of the different SV calling methods over the 3,278 (8.76%) mid-size deletions (300-1000 bp) (see **Supplementary Table 1** for details). Parliament2 had the second-highest recall (83.20%) and the highest precision (96.49%). Only MetaSV had a marginally higher recall (83.81%). Again, Parliament2 showed the highest F1 score (89.35%), followed by Manta (86.65%). For deletions larger than 1 kbp (**Figure 1c**; see **Supplementary Table 1** for details) only 1,614 (4.31%) of the gold standard, MetaSV showed the highest F1 score (89.83%) closely followed by Parliament2 (87.89%), both driven by their high precision scores of 91.92% and 91.59%, respectively. Across all size regimes for deletions, Parliament2 achieved by far the highest F1 score (average: 81.73%) followed by Manta (77.31%), MetaSV (68.06%), Delly (65.20%), Breakseq (63.28%), Breakdancer (58.78%), Lumpy (49.96%), and CNVnator (11.12%).

We further assessed the recall and precision across insertions for Parliament2 over the HG002 sample. Across all callers, Parliament2 yielded a high precision score (94.13%) and a recall score consistent with GIAB's incorporation of long-read technologies (19.21%).

To avoid a biased benchmarking, we further benchmarked Parliament2 against three assembly-based SV call sets [20] from non-Caucasians to highlight Parliament2's versatility. **Supplementary Figure 2** shows the results split for deletions and insertions, as these are the only SVs previously reported. Parliament2 again achieves the highest recall and precision for insertions and deletions. Nevertheless, as expected, the recall for insertions is reduced compared to deletions given the limitations of short-read based insertion detection.

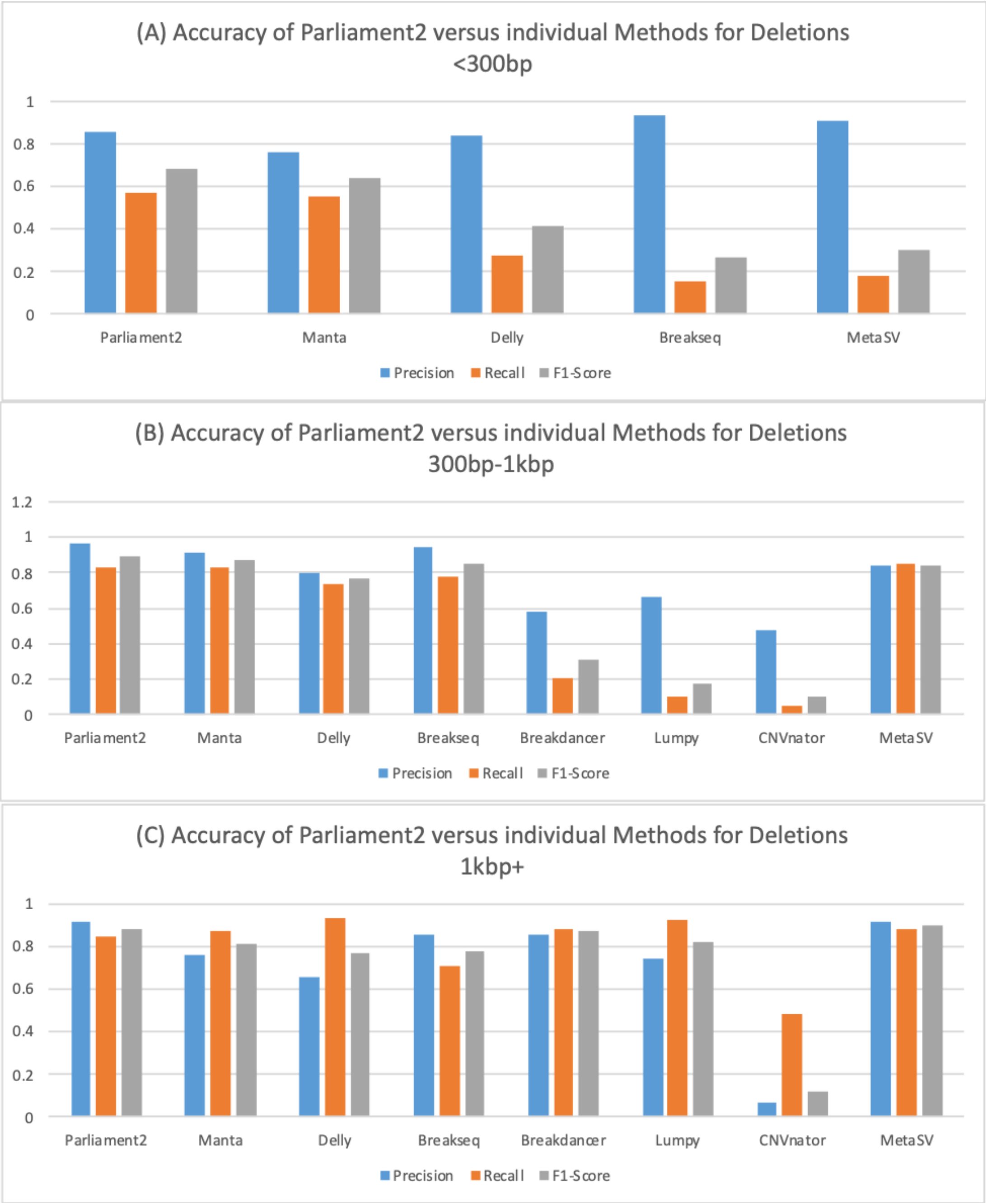

Figure 1*. Accuracy comparison for Parliament2 based on GIAB v0.6 deletion call set for different size regimes of deletions. (A) less than 300 bp, (B) between 300 bp and 1 kbp, and (C) larger than 1 kbp. The order of methods in each graph is sorted such that methods with higher F1 scores are located to the left. The efficacies of individual methods vary between size ranges.*

Compute Efficiency

Runtime and computational efficiency are essential to scalability and cost reduction. The SV callers used by Parliament2 fall into three parallelization classes: native multi-threading (Breakseq, Manta); native parallelization by chromosome (CNVnator, Breakdancer); and those lacking either (Delly, Lumpy). Upon execution, Parliament2 immediately executes Breakseq and Manta with multiple threads, splits the input BAM by chromosome, and initiates runs on the remaining callers. For the 35x HG002 BAM, this strategy

reduced the runtime for Lumpy from 6.45 hours to 0.45 hours and for Delly from 8.52 hours to 0.67 hours on a 16-core machine.

The parallelization across multiple programs leads to a reduction in runtime by achieving higher overall machine utilization of resources (**Figure 2**). In local and cloud environments, this optimization translates to reductions of cost, CPU utilization, and wall time.

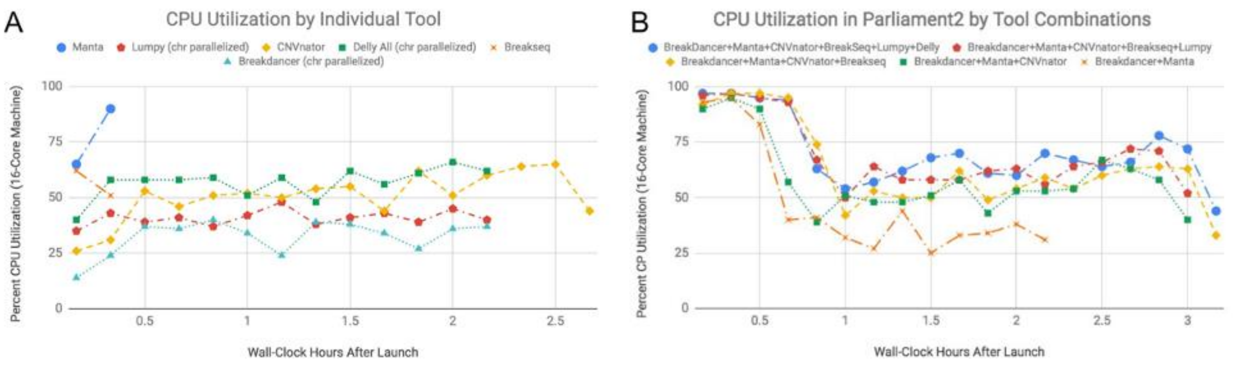

Figure 2. *Concurrent execution of multiple tools in Parliament2 increases resource utilization. (A) Percent of total CPU utilization on a 16-core machine executing Parliament2 and running only an individual tool. Each line terminates when the program finishes executing. (B) Resource utilization when running combinations of methods simultaneously within Parliament2.*

Consensus Quality Scores

One oft-discussed problem for short-read based SV calling is low sensitivity and high false discovery rates [5,6]. This challenge is exacerbated by the variety of SV types and sizes and the applicability of various methods to each SV class. The different performances of individual methods (see above) highlight the potential of a consensus approach stratified by size and event type. Without such a distinction, accuracy assessments are dominated by the more numerous small events, potentially under-reporting rare but impactful gene-sized events.

We analyzed the contribution of each Parliament2 caller to the overall precision. **Figure 3A** describes how each combination of SV methods contributes to recall performance. The precision of SV calls obtained by a single individual method ranges from 8% with CNVnator to 91% for Breakseq. However, when an SV call is supported by multiple methods, precision can reach 100% independent of the size regime (**Figure 3A, 3B**). The combination of CNVnator and BreakSeq is the minimum set of SV callers to reach 100% precision. Although CNVnator has the lowest precision performance (8%), it is included in every set that reaches a 100% recall rate. Thus, while deletions discovered only by CNVnator have low precision, a deletion call from CNVnator and at least one other method provides high precision. While only a few methods (Breakseq, Manta, and Delly) detect insertions, they are generally precise (98%-100%). **Supplementary Figure 1** shows the precision of the individual SV caller and their combinations for insertions.

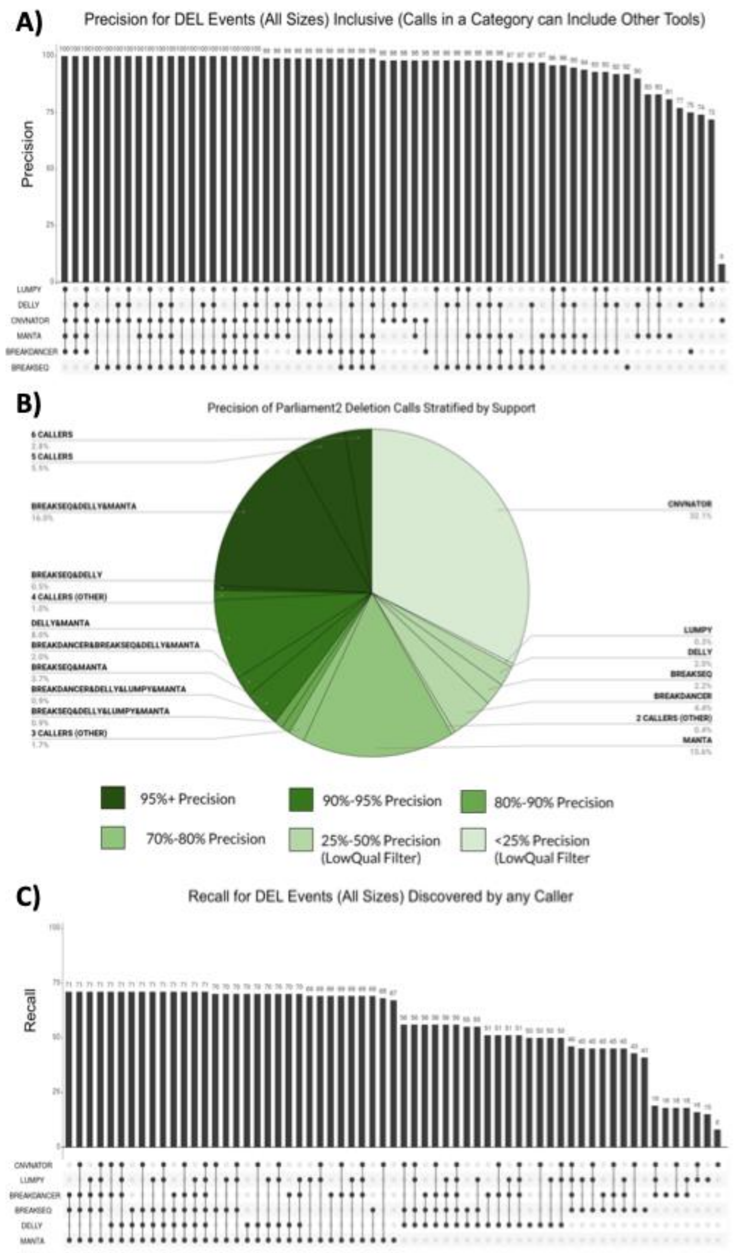

Figure 3: *Assessment of constituent SV calling methods based on the deletion call set from GIAB v0.6. A) Measured precision for the different method combinations. The precision ranges from 5% (CNVnator) up to 100% for various combinations. B) Contributions of the individual SV callers and their combinations to the total number of calls (percent label) and their relative precision (color-coded by shade of green). C) Measured recall for individual methods and their combinations ranging from 8% (CNVnator) to 71% for various combinations.*

Figure 3C details the recall rates of individual SV callers and their combinations. The highest recall rate (71%) is achieved by a combination of multiple callers. This value is surprisingly high given that the truth set includes data from multiple long-read technologies and SVs that were only obtained by long-read sequencing and assembly. Manta is included in all of the combinations that reached a high recall value for deletions. For insertions, the overall recall is drastically reduced to 17% using a combination of Manta (15%), Delly (3%), and BreakSeq (2%) (**Supplementary Figure 1**).

Based on these observations, we generated a ruleset based on GIAB deletion and insertion calls using the supporting callers, type of event, and size of event (for deletions), assuming the individual SV callers show similar metrics in other types of SVs. This ruleset is then applied to assign quality values to each of the reported SV calls. Parliament2 expresses the call quality as a Phred-encoded value within the final consensus VCF. These scores are based on the precision results from GIAB for each combination of supporting callers, the type of the event (deletion or insertion), and the size category of the event (50-300 bp, 300-1000 bp, >1 kbp). This quality value allows investigators to set thresholds to achieve the trade-off between precision and recall that is desired for their use cases or to prioritize events based on how likely they are to be true events. **Supplementary Figure 1** shows the quality value based on caller support and the SVs type and size. These quality values enable Parliament2 to obtain a balanced performance for recall rate and precision resulting in the highest F1 scores (**Figure 1a-c**) across multiple size regimes. The same ruleset is also applied to other SV types for which we lacked GIAB benchmark data (e.g. inversions).

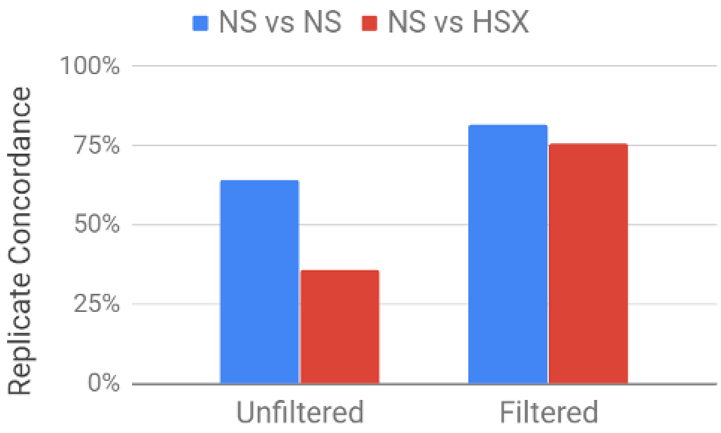

Figure 4: Parliament2 HG002 concordance across NovaSeq (NS) and HiSeqX (HSX) before and after quality filtering.

Inter-Platform Concordance

Large collaborative projects aggregate heterogeneous data across different sequencing centers, chemistry versions, and short-read platforms (e.g. HiSeq X and NovaSeq). Given the inferential nature of SV detection from short reads, SV methods are particularly susceptible to batch effects. Therefore, we have characterized Parliament2 using HiSeq X and NovaSeq sequencing runs, including the HiSeq X data described above and four NovaSeq HG002 replicates each downsampled to 35x coverage and mapped to the hs37d5 reference. These 35x NovaSeq replicates showed similar precision (83.0%) and recall (69.35%) compared to the HiSeq X (81.7% and 70.7%, respectively). Increasing coverage to 50x for all samples across both platforms changed these values by <5% (see **Supplementary Table 2** for details), indicating the robustness of evaluating both platforms at 35x. The unfiltered concordance values, corresponding to all raw Parliament2 consensus calls, indicate low inter-platform consistency, which would likely drive batch effects in mixed-platform sample sets (**Figure 4**). After filtering for Parliament2 events with a quality value greater than 3, inter- and intra-platform concordances increase to similar levels, suggesting both an increase in quality and mitigation of platform batch effects (**Figure 4**).

1000 Genomes Project SVs for GRCh38

The 1000 Genomes Project (1KGP) is a valuable resource of high-confidence SV calls across a large sample set (2,691 samples) mapped to GRCh37. However, since the introduction of GRCh38 [21], many

large-scale whole-genome programs (e.g. TOPMed, All of Us) have adopted this standard. To demonstrate the scalability of Parliament2 for large datasets and to create a community resource, we applied Parliament2 to the 2,691 1KGP WGS samples mapped to GRCh38 [21]. Although the 1KGP samples have been remapped to GRCh38 [21,22], we are not aware of a comprehensive set of SVs on these data and reference sets.

The computational requirements were modest in comparison to other familiar applications, and the entire SV calling was completed in one day of wall clock time, using only 63,720 CPU-hours (on average 24 core-hours per sample). For reference, that amount of compute is approximately equivalent to running GATK4 on 220 WGS samples at 35x coverage. This effort created SVs calls for each of Breakdancer, CNVnator, Delly, Lumpy, and Manta, as well as SVTyped files of each and consensus Parliament2 calls. **Figure 5A+B** shows the results for these call sets.

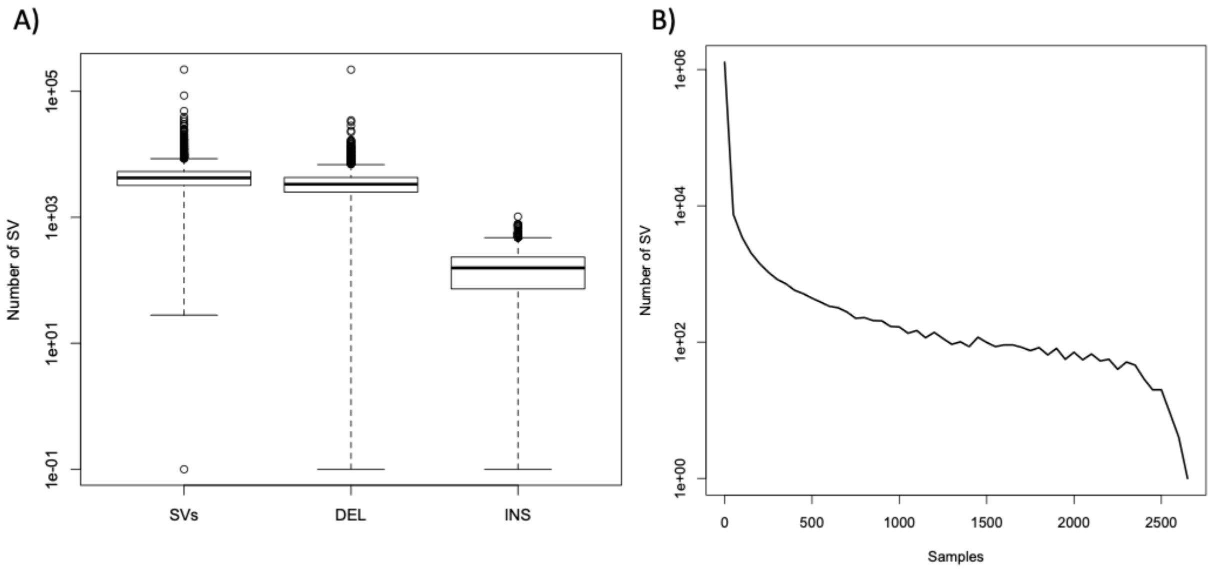

Figure 5. *Population distribution of SV calls produced by Parliament2 for the 1000Genomes Phase3. A) Number of SV, deletions, and insertions across the 2506 samples. B) Allele frequency across all SV at log scale showing an expected distribution of a high number of rare SV and very few common SV or fixed SV.*

Figure 5A shows the distribution of SV inferred per sample across all 1KGP samples. In total, there are 88,404 deletions larger than 50 bp discovered in this set and 30,479 inversion events. There were only 619 insertions discovered, possibly reflecting that it is more difficult to detect an insertion in these low coverage data. The number of calls per sample was generally lower than observed for the high-coverage WGS samples investigated in the prior benchmarks (**Figure 5A**). Additionally, for certain samples, some of the callers did not generate any output, possibly due to low sequence coverage of the samples. Nevertheless, the allele frequency profile looks as expected (**Figure 5B**) as it reassembles a high amount of private SV vs. a much lower amount of common SV. In addition, we observed only minor fixated alleles as the samples span a large set of different populations. These SV calls will provide a resource to understand SVs called on GRCh38 relative to the multiple ethnicities captured in 1KGP and to understand how each of these tools interacts with lower coverage data.

Interoperability of Parliament2 using WDL

A newer version of Parliament2, which uses the same tools and principles discussed earlier, is open-source and available as a code base (https://github.com/slzarate/parliament2). This version increases the

interoperability of Parliament2. Instead of implementing all tools on a single Docker image, this workflow uses the Workflow Development Language (WDL) to run each parallelized SV caller as a task, with separate Docker images implemented for each tool. This can then be imported to different cloud platforms, including DNAnexus and AnVIL/Terra (http://anvilproject.org), as well as any environment configured to run Cromwell, such as HPC clusters. As a result, this version of Parliament2 enables a better adoption to other infrastructures and is more modularly implemented.

This WDL workflow version runs in 4.52 hours (wall time) compared to the main version (3.43 hours) on the same 35x coverage BAM aligned to the hs37d5 reference genome used for benchmarking in previous sections. The increased runtime is likely due to both the increased I/O required for spinning up different machines for each task and the fact that these machines had fewer than 16 cores.

Furthermore, the WDL version of Parliament2 upgrades several tools. Due to these upgrades, the SV call set produced is modestly different than that generated by the original version of Parliament2 benchmarked here, though the overlap is quite high (87.37% of the WDL output overlaps with the original). Among insertions and deletions, which we benchmark above, overlap is 84.87% for insertions, 90.95% for deletions, and 90.23% for both insertions and deletions combined. We inspected the differences between the call sets and found they were chiefly due to borderline calls that were just above or below the thresholds of the tools used. This version also integrates Jasmine [23] as an alternative to SURVIVOR for merging SVs.

In this manuscript, we presented Parliament2 for identifying SV at scale for short-read data sets. The Parliament2 optimized consensus approach addresses the accuracy and compute challenges of calling SVs from short-read sequence data at scale. Leveraging consensus calling for event discovery and quality assessment, Parliament2 achieves a higher overall accuracy (F1 score against GIAB HG002 SVs) than any constituent method without compromising efficiency, providing robust SV calling across multiple platforms. Parliament2 is unique in its capability to identify multiple classes of SVs in an easily scaled manner, enabling efficient computation on a single sample (~3 hours) or on thousands of samples. Within one day of Parliament2 compute, we have generated the first comprehensive SV set for the 1KGP samples on GRCh38, a publicly available resource (see **Supplement**).

Parliament2 specializes relative to MetaSV in two key ways. First, Parliament2 is optimized for scalability, not requiring an expert user to launch multiple SV callers, the results of which need to be combined later (e.g. SURVIVOR, MetaSV). This leads to a faster and more efficient execution over thousands of samples. Parliament2 pre-installs these programs, configures them to speed up the processing, and utilizes a trained quality value to provide extra information about the reliability of the SV calls. Second, MetaSV does not provide a full workflow and includes costly assembly steps that result in high computational costs over multiple samples. Nevertheless, these enable MetaSV to report sequence-resolved insertion calls, while Parliament2 can only produce the sequence resolution if the individual method that called the event produced it. Still, this complicates the execution of MetaSV over multiple hundred to thousands of samples required for larger cohorts.

SV calling accuracy, however, remains an open challenge. F1 scores for best-in-class small variant callers routinely exceed 99%, and even higher standards are required for clinical reporting. As SV methods improve, the Parliament2 infrastructure can be easily adapted to incorporate new methods (e.g. graph-based references and rapid local assembly) and SV callers, especially those that target specific SV types such as mobile element insertions and variable nucleotide tandem repeats, to determine the optimal consensus strategy. Such improvement will be accelerated by broader and deeper high-confidence SVs

from long-range data across more samples and ethnicities against which SV methods such as Parliament2 can be trained.

Methods

Parliament2 Implementation

The code for Parliament2 is available at a GitHub repository (https://github.com/dnanexus/parliament2) with an open-source (Apache-2.0) license at the 1.0.7 version (commit 97517b1a22104a3e0a0966a79c3b5556fde8a89d). Execution of Parliament2 done by running v1.0.7 of the Parliament2 DNAnexus app (app-FJ8Fj88054JxXFygKvFqQ39j), which is publicly available to run by any user on DNAnexus. This app runs a Docker image built directly from the GitHub repository, which is available on DockerHub. Executions of the app with user-provided input for tool combinations specify the parameter flags to the Docker image to include or exclude the desired tools.

Parliament2 Implementation (WDL)

The WDL version of Parliament2 is available at a GitHub repository (https://github.com/slzarate/parliament2) with an open-source (Apache-2.0) license at the 0.0.1 version (commit ed86345740f029093365f8a3b0d99f9cb153c9ed). For these tests, the execution of Parliament2 was done by importing this version of the WDL file into DNAnexus, which automatically converts the WDL file into a native workflow. This WDL file specifies Docker image versions, which allow for this code to be easily replicated. The Docker images are available on DockerHub and are built using the code available on the GitHub repository.

Input WGS Data Used for Timing and Accuracy Benchmarks

Timing statistics and resource utilization were determined by executing the Parliament2 app on a 35X WGS sample for HG002 that was made by random downsampling the 50X PCR-Free HG002 HiSeqX sample generated for the Challenge set of the PrecisionFDA Truth Challenge.

Timing for individual tools and Parliament2 combinations

All timing calculations are run on a c3.4xlarge AWS instance (16-core, 30GB RAM, 320GB disk). To calculate the runtime and resource utilization of individual components, the Parliament2 app was launched with the desired tool or tool combinations. DNAnexus apps write an entry of machine resources (CPU percent, RAM, and disk utilization) every 10 minutes to a job log that also contains the stdout and stderr outputs for job execution. All info log entries of this after the stderr line for program execution up until the SVTyper step (which indicates completion of all jobs) were taken to determine the resource plots over time.

The logs for these jobs are available at:
https://github.com/dnanexus/parliament2/tree/master/benchmarking_data/dx_job_logs

Accuracy comparisons

Accuracy comparisons are performed using Truvari (https://github.com/spiralgenetics/truvari) with the following execution: truvari.py -b GIAB_DEL0.6.vcf.gz -c <parliament_output> -o <output_directory --passonly --includebed GIAB_0.6.bed --pctsim=0 -r 2000 –giabreport

To determine accuracy for size ranges, -s <lower_size> and -S <upper_size> were used. The deletion truth set was taken by extracting SVTYPE=DEL from the v0.6 truth set. The insertion truth set was taken similarly by extracting SVTYPE=INS.

The Genome in a Bottle data for v0.6 truth set is at:
ftp://ftp-trace.ncbi.nlm.nih.gov/giab/ftp/data/AshkenazimTrio/analysis/NIST_SVs_Integration_v0.6/

Tool Versions

The individual tools that comprise Parliament2 run the following versions of each program:
Breakdancer: [v1.4.3]
(https://github.com/genome/breakdancer/releases/tag/v1.4.3)
BreakSeq2:[v2.2]
(http://bioinform.github.io/breakseq2/)
CNVnator:[v0.3.3]
(https://github.com/abyzovlab/CNVnator/commit/de012f2bccfd4e11e84cf685b19fc138115f2d0d)
Delly:[v0.7.2]
(https://github.com/dellytools/delly/releases/tag/v0.7.2)
Lumpy: [v0.2.13]
(https://github.com/arq5x/lumpy-sv/commit/f466f61e02680796192b055e4c084fbb23dcc692)
Manta: [v1.4.0]
(https://anaconda.org/bioconda/manta)
SURVIVOR: [v1.0.3]
(https://github.com/fritzsedlazeck/SURVIVOR/commit/7c7731d71fa1cba017f470895fb3ef55f2812067)
SVTyper: [v0.7.0]
(https://github.com/hall-lab/svtyper/commit/5fc30763fd3025793ee712a563de800c010f6bea)
Svviz: [v1.5.2]
(https://github.com/svviz/svviz/commit/84acefa13bf0d4ad6e7e0f1d058aed6f16681142)

The individual tools that comprise the WDL version of Parliament2 run the following versions of each program:
Breakdancer: [v1.4.3]
(https://github.com/genome/breakdancer/releases/tag/v1.4.3)
BreakSeq2:[v2.2]
(https://anaconda.org/bioconda/breakseq2/)
CNVnator:[v0.3.3]
(https://github.com/abyzovlab/CNVnator/releases/tag/v0.4.1)
Delly:[v0.8.3]
(https://anaconda.org/bioconda/delly)
Lumpy: [v0.3.0]
(https://anaconda.org/bioconda/lumpy-sv)
Manta: [v1.4.0]
(https://anaconda.org/bioconda/manta)
SURVIVOR: [v1.0.7]
(https://github.com/fritzsedlazeck/SURVIVOR/commit/1d1d33b016dbf818b1678a27dee3d3de7f0fda0b)
JASMINE: [v1.0.6]
(https://github.com/mkirsche/Jasmine/releases/tag/v1.0.6)
SVTyper: [v0.7.0]
(https://anaconda.org/bioconda/svtyper)
Svviz: [v1.6.2]
(https://anaconda.org/bioconda/svviz)

Availability of supporting source code and requirements

Project name: Parliament2
Project home page: https://github.com/dnanexus/parliament2
Operating system(s): Linux
Programming language: Bash/Python/C++
Other requirements: Docker

Availability of supporting data

Benchmark output:

All benchmark results of all the programs can be found:
https://github.com/dnanexus/parliament2/tree/master/benchmarking_data/hg002_benchmarks

1000 genome download links for the following resources are:

A project-level VCF of all PASS and unfiltered variants in any sample:
https://github.com/dnanexus/parliament2/tree/master/benchmarking_data/1000_genomes
The VCF output of Parliament2:
https://github.com/dnanexus/parliament2/tree/master/benchmarking_data/hg002_benchmarks
The individual caller files for Breakdancer, BreakseqCNVnator, Delly, Lumpy, and Manta are available at:
https://github.com/dnanexus/parliament2/tree/master/benchmarking_data/hg002_benchmarks/sv_caller_outputs

Competing interests

This work was conducted when SZ and AC were employed at DNAnexus. Neither SZ nor AC are currently employed by DNAnexus and do not have financial conflicts to disclose. FJS has multiple sponsored travels from Oxford Nanopore and PacBio and is the recipient of the 2018 SMRT PacBio grant.

Funding

DNAnexus contributed computational resources and funding for personnel in this project. This work has been supported by NHGRI Centers for Common Disease Genomics (5UM1HG008898-02), ANVIL (5U24HG010263), and CCDG (UM1 HG008898). The funding body did not play any roles in the design of the study and collection, analysis, and interpretation of data and in writing the manuscript.

Authors' contributions

SZ and AC implemented Parliament2 as a Docker image and a DNAnexus app. SZ implemented Parliament2 as a WDL workflow. FJS implemented SURVIVOR and adopted it for Parliament2. SZ, AC, MM, OK, GJ, WJS, MCS, EB, RAG, and FJS contributed to writing the manuscript and study design.

Acknowledgments

We would like to thank John Didion and Mike Lin for helpful discussion regarding the WDL implementation of Parliament2.

References

1. Feuk L, Carson AR, Scherer SW. Structural variation in the human genome. Nature Reviews Genetics. 2006. p. 85–97. Available from: http://dx.doi.org/10.1038/nrg1767

2. Lupski JR. Structural variation mutagenesis of the human genome: Impact on disease and evolution. Environmental and Molecular Mutagenesis. 2015;56:419–36.

3. Mahmoud M, Gobet N, Cruz-Dávalos DI, Mounier N, Dessimoz C, Sedlazeck FJ. Structural variant calling: the long and the short of it. Genome Biology. 2019;20:246.

4. Alkan C, Coe BP, Eichler EE. Genome structural variation discovery and genotyping. Nature Reviews Genetics. 2011. p. 363–76. Available from: http://dx.doi.org/10.1038/nrg2958

5. Sedlazeck FJ, Rescheneder P, Smolka M, Fang H, Nattestad M, von Haeseler A, et al. Accurate detection of complex structural variations using single-molecule sequencing. Nature Methods. 2018;15:461–8.

6. Chaisson MJP, Huddleston J, Dennis MY, Sudmant PH, Malig M, Hormozdiari F, et al. Resolving the complexity of the human genome using single-molecule sequencing. Nature. 2015;517:608–11.

7. Chen K, Wallis JW, McLellan MD, Larson DE, Kalicki JM, Pohl CS, et al. BreakDancer: an algorithm for high-resolution mapping of genomic structural variation. Nature Methods. 2009;6:677–81.

8. Abyzov A, Urban AE, Snyder M, Gerstein M. CNVnator: an approach to discover, genotype, and characterize typical and atypical CNVs from family and population genome sequencing. Genome Research. 2011;21:974–84.

9. Wang J, Mullighan CG, Easton J, Roberts S, Heatley SL, Ma J, et al. CREST maps somatic structural variation in cancer genomes with base-pair resolution. Nature Methods. 2011;8:652–4.

10. Rausch T, Zichner T, Schlattl A, Stütz AM, Benes V, Korbel JO. DELLY: structural variant discovery by integrated paired-end and split-read analysis. Bioinformatics. 2012;28:i333–9.

11. Layer RM, Chiang C, Quinlan AR, Hall IM. LUMPY: a probabilistic framework for structural variant discovery. Genome Biology. 2014;15:R84.

12. Chen X, Schulz-Trieglaff O, Shaw R, Barnes B, Schlesinger F, Källberg M, et al. Manta: rapid detection of structural variants and indels for germline and cancer sequencing applications. Bioinformatics. 2016;32:1220–2.

13. Ye K, Schulz MH, Long Q, Apweiler R, Ning Z. Pindel: a pattern growth approach to detect break points of large deletions and medium sized insertions from paired-end short reads. Bioinformatics. 2009. p. 2865–71. Available from: http://dx.doi.org/10.1093/bioinformatics/btp394

14. Mohiyuddin M, Mu JC, Li J, Bani Asadi N, Gerstein MB, Abyzov A, et al. MetaSV: an accurate and integrative structural-variant caller for next generation sequencing. Bioinformatics. 2015;31:2741–4.

15. English AC, Salerno WJ, Hampton OA, Gonzaga-Jauregui C, Ambreth S, Ritter DI, et al. Assessing structural variation in a personal genome—towards a human reference diploid genome. BMC Genomics. 2015. Available from: http://dx.doi.org/10.1186/s12864-015-1479-3

16. Jeffares DC, Jolly C, Hoti M, Speed D, Shaw L, Rallis C, et al. Transient structural variations have strong effects on quantitative traits and reproductive isolation in fission yeast. Nature Communications. 2017. Available from: http://dx.doi.org/10.1038/ncomms14061

17. Chiang C, Layer RM, Faust GG, Lindberg MR, Rose DB, Garrison EP, et al. SpeedSeq: ultra-fast personal genome analysis and interpretation. Nature Methods. 2015;12:966–8.

18. Spies N, Zook JM, Salit M, Sidow A. svviz: a read viewer for validating structural variants. Bioinformatics. 2015;31:3994–6.

19. Zook JM, Hansen NF, Olson ND, Chapman L, Mullikin JC, Xiao C, et al. A robust benchmark for detection of germline large deletions and insertions. Nature Biotechnology. 2020; Available from: http://dx.doi.org/10.1038/s41587-020-0538-8

20. Chaisson MJP, Sanders AD, Zhao X, Malhotra A, Porubsky D, Rausch T, et al. Multi-platform discovery of haplotype-resolved structural variation in human genomes. Nature Communications. 2019;10:1784.

21. Sudmant PH, Rausch T, Gardner EJ, Handsaker RE, Abyzov A, Huddleston J, et al. An integrated map of structural variation in 2,504 human genomes. Nature. 2015;526:75–81.

22. Zheng-Bradley X, Streeter I, Fairley S, Richardson D, Clarke L, Flicek P, et al. Alignment of 1000 Genomes Project reads to reference assembly GRCh38. Gigascience. 2017;6:1–8.

23. Alonge M, Wang X, Benoit M, Soyk S, Pereira L, Zhang L, et al. Major Impacts of Widespread Structural Variation on Gene Expression and Crop Improvement in Tomato. Cell. 2020;182:145–61.e23.

Supplementary

Figure S1. Quality values assigned by Parliament2 to SV events of various types, sizes, and support. Parliament2 assigns a quality value to each event based on the precision observed in comparisons with the GIAB v0.6 truth set. In the above figure, the event type (deletion or insertion) and size determine the color code. The maximum QV assigned is 40, even if the precision of the subset is higher. Only categories with more than two calls are included.v

Figure S2. Benchmark comparison of Parliament2 on non caucasian samples. We have benchmarked Parliament2 SV calls (insertion and deletions) across three non-caucasian samples (HG00514, HG00733, NA19240) that have been previously characterized by de novo assembly including multiple sequencing technologies. Overall Parliament2 shows a high concordance with the deletion calls to Chaisson et. al. with only very few calls that are private to Parliament2. For insertions, however, the recall ability is reduced due to limitations from the short reads.

Supplement Tables

Click here to access/download
Supplementary Material
Parliament2_tables.xlsx

Supplementary Figures

Click here to access/download
Supplementary Material
Figures_Parliament2.pptx

We would like to thank both reviewers for their constructive feedback, which has improved the manuscript and thus the presentation of Parliament2. We have added benchmarks for three additional non-Caucasian samples where a highly curated set of SV based on an assembly approach was available. Furthermore, we have also now implemented Parliament2 as a WDL workflow, which allows for a more modular execution and easier maintenance. Due to the implementation in WDL, Parliament2 is now also hosted on the NIH/NHGRI AnVIL platform (http://anvilproject.org), which will help many scientists to explore their and others' data sets for structural variation detection. As requested, we have also included insertions into our benchmarks, which again reflected a high precision for Parliament2 and the highest recall among the short read SV callers assessed here.

We have marked the responses in blue to each individual question/concern of the reviewers and highlighted the changes in the manuscript also in blue.

Reviewer #1: Parliament2
Overall
This manuscript represents an cloud-based ensemble method that incorporate multiple state-of-the-art algorithms for SV discovery at high sensitivity, and implemented a series of quality control(QC) steps to ensure the specificity. Improved performance were observed when compared against individual algorithms, at the cost of higher computing costs / resources. While informative and timely, there are major flaws in the study design, such as wrong assumptions being adopted while calculating quality score, or conflict information delivered by the text and Figure 5. Moreover, several places in this manuscript deliver confusing information, and the author could use more help in professional writing.

Major:

1. Conclusion in the Abstract (page2): Is Parlimament2 designed for SV discovery of single sample, or for group of samples?

Parliament2 is calling structural variation per sample and delivers one merged and genotyped VCF file. Nevertheless, Parliament2 is designed for speed and accuracy to enable large scale SV calling, and has already been applied to the 1000 Genomes, TopMed, and CCDG data sets, which together comprise around 260,000+ human genome samples.

2. Last sentence of the first paragraph under "Findings" (page2)
"Even best-in-class methods can fail to capture the majority of SVs (30% to 70% sensitivity) and often return a high false discovery rate, especially for insertion and inversion events [5,6]."

   a. The author should define 'high false positive rate', by showing the actual FDR rate in this sentence

   This was extracted from previous papers that were cited. Other papers, such as those produced by GIAB, the 1000 Genomes Project etc., have shown similar results. The false discovery rate ranges based on the SV type and size ranges the studies investigate for short reads.

   b. The author should also define 'best-in-class methods' , which specific algorithms are referred to here?

   We were trying to reflect on the most commonly used/well-cited SV callers such as Delly, Lumpy, and Manta (cited 1011, 657, and 432 times, respectively, and top performers in several benchmarks such as https://genomebiology.biomedcentral.com/articles/10.1186/s13059-019-

1720-5). However, this is directed to give the reader an overview of the current challenges in the field. See above.

c. The two publications cited here both focused on SV discovery from long reads whole genome sequencing technology, which is a different sequencing platform and is not really comparable to any short read paired end sequencing methods. moreover, neither of the cited paper mentioned benchmark results of SVs from short read sequencing methods against long read methods, I don't think these two publications can be cited to support this sentence.

Multiple papers report long-read sequencing as state-of-the-art to obtain the most comprehensive detection of structural variations. We don't agree with the reviewer that these SV calls cannot be compared to short reads, as this has been done multiple times over the past years (e.g. HGSV work, GIAB, etc.).

3. First sentence of the second paragraph under "Findings" (page2) "Common SV detection methods, including Breakdancer [7], CNVnator [8], Crest [9], Delly [10], Lumpy [11], Manta [11,12], and Pindel [13]," 'Common SV detection methods' reads confusing here, it can be interpret as either 'commonly used SV detection methods' or 'SV methods used to detect common SVs among population', the author should clarify;

We thank the reviewer for pointing this out and clarified it based on the suggestion: 'Commonly used SV detection methods'.

4. Second paragraph on page3 "Parliament2 executes any combination of Breakdancer, Breakseq, CNVnator, Delly, Lumpy, and Manta to generate candidate SV events" Does Parliament2 support any other algorithms? If yes, how? If no, why isn't any MEI specific algorithms included? None of these listed algorithms have shown comparable performance to MELT, it should be supported for a full scale SV discovery.

Parliament2 is currently highly optimized in packaging the 6 SV callers to optimize runtime and utilization for the compute note. Thus, it's currently not easily possible to include other callers. Nevertheless, since the review, we have extended Parliament2 with a new workflow engine that enables it to be run on ANVIL and other infrastructures efficiently that would more easily allow for such an adjustment. MELT and MEI are currently not included in Parliament2.

5. The section "Accuracy assessments for Parliament2 based on real data"

a. A brief description of the GIAB sample should be described here, as the authors should not expect audiences to have read the Zook et al 2019.

We have added this in the main text: "The GIAB SV truth set is based on HG002, a male Ashkenazi Jewish sample utilizing multiple technologies and manual vetting of the SV."

b. Parliament2 outputs SVs of different types, including deletions, duplications, insertions, inversions and translocations, however, only deletions are benchmarked. The author should also have provided the performance comparison of insertions. Focusing on deletions could be biased for algorithms that are specifically designed for deletions.

We have now extended this to insertion calls. The precision is very good (94.13%), and the sensitivity is -- as expected -- reduced compared to long-read approaches at 19.21%. We have

also now benchmarked these for 3 other samples (see below/ Supplementary Figure 2). Unfortunately, there is no gold standard SV set available for rearrangements.

c. Why are lumpy and Breakdancer not included in comparison of deletions <300bp? Both methods should have generated good amount of deletions in this size range.

In our comparison, they did not report smaller deletions than this size. For Lumpy, this is reported in an issue where one needs to adapt the run just to recover the sizes of <500bp https://github.com/arq5x/lumpy-sv/issues/68. For Breakdancer, we had a similar problem; based on their GitHub page, they recommend using PINDEL for smaller deletions("We recommend using Pindel to detect intermediate size indels (10-80 bp)."): https://github.com/genome/breakdancer

d. The author briefly described computational cost of the Parliament2 in one sentence: "Parliament2 ran in 3.43 hours (wall time) on a 16-core machine from a 35x coverage BAM aligned to the hs37d5 reference genome." But I don't see why this is necessary as there's a whole paragraph discussing the computational cost right after this paragraph. Moreover, this sentence is confusing, is only one sample used for the comparison here? Or is 3.43 hours averaged across multiple samples? If so, how many? The authors should have provided more information about the samples used for this comparison

Reviewers and readers are often interested in the overall runtime when interpreting the presented results. As this is just one sentence we think it adds to the overall picture. Later, in the results presented over the runtime improvements, we show how we could achieve this and give the details of improvement over each component.

e. Define F1 score, and explain why how does this score represents the performance of each methods;

We have added a comment in the main text. In brief: F1 score (also F-score or F-measure) is a common metric in benchmark studies, as it combines the recall and precision (https://en.wikipedia.org/wiki/F1_score). Thus, a method that recalls everything but also has multiple errors will not perform well, and neither will a method that correctly calls only 1 SV.

6. "Compute Efficiency", page 6

a. The author should have provided estimation of the overall run time of Parliament2 on a 35X genome.

We reported this: "Parliament2 ran in 3.43 hours (wall time) on a 16-core machine from a 35x coverage BAM aligned to the hs37d5 reference genome."

b. Direct comparison of computing cost in terms of overall CPU hours on fixed number of cores should be provided between Parliament2 and other individual methods. The author indicated better usage of computing source were achieved through parallelization, however the overall cost of running multiple algorithms, intergrade and quality control should still be higher than each individual algorithms. The comparison of overall cost can get audience a clear idea of the tradeoff between computing cost and increased performance when deciding on SV calling methods.

Figure 2 shows the comparison between Parliament2 and the individual methods which compose it and were specifically parallelized for Parliament2. However, with Parliament2, the results for all methods are produced at the same time. The point here was that one needs to rent a compute node (e.g. from Amazon) that has a certain set of cores and memory. Parliament2 is not per se faster than any single SV caller benchmarked, but is faster than a combination of the SV callers and optimizes some of them by splitting up the data set. We were able to significantly speed up e.g. Lumpy, but the real improved design was in packaging the individual SV callers together so that the compute node is fully utilized.

7. "Consensus Quality Scores", page 6

a. "One oft-discussed problem for short-read based SV calling is low sensitivity and high false discovery rates [5,6]." The author commented on the performance of current short read based SV discovery method, by citing two publications that focused on SV discovery from single molecule long reads sequencing technologies. However, Parliament2 is also a short read based method, so I wouldn't expect it to overcome all the challenges of short reads and achieve comparable performance to long reads, neither are there any such evidences provided in this manuscript. So I cannot see why the two publications were cited here and how they can be used to support the point.

   We are not claiming that we solve all short read based SV calling problems. One of the problems here is conceptually about mismappings or worse representations of SV based on the short reads. This sentence is the start of a section and again describes, similar to your previous critique, the current state and challenges. Nevertheless, our benchmarks indicate that by combining multiple SV callers,Parliament2 does indeed achieve a high sensitivity while maintaining a high precision and thus achieve a high F-score.

b. "Based on these observations, we generated a ruleset based on GIAB deletion calls assuming the individual SV callers show similar metrics in other types of SVs." This assumption is not true, each type of SV should be analyzed independently.

   We agree and even state that this is not optimal. At this time, however, only deletions and insertions are available from highly curated SV call sets. Parliament2 has separate quality calibrations for deletions and insertions, based on which callers support and the size and type of event (see: https://github.com/dnanexus/parliament2/blob/master/resources/all.phred.txt) for the full list of PHRED qualities. As deletions are more frequently called by short read based SV callers such as Parliament2, and more of the methods used call deletions than insertion events, the quality distributions for deletions are more diverse and informative than those for insertions.

   We have modified this sentence to read "Based on these observations, we generated a ruleset based on GIAB deletion and insertion calls using the supporting callers, type of event, and size of event (for deletions), assuming the individual SV callers show similar metrics in other types of SVs"

c. If GIAB callset cannot provide enough benchmark data to derive consensus quality scores for SV types other than deletions and insertions(as the author stated, "The same ruleset is also applied to other SV types for which we lacked GIAB benchmark data (e.g. inversions)."), the author should seek studies for gold standard SV callset such as Chaisson et al(2019, Nat Commun), which provided SV calls across different types, including inversions.

We have downloaded the VCF files from dbVar and investigated the call sets. The VCF reports mainly insertions and deletions with only very few variants of LOC type, which are not well-defined. We have added benchmarks to the three samples for insertions and deletions in Supplementary Figure 3 and mentioned the results in the main text.

8. "Inter-Platform Concordance"
    a. "Increasing coverage to 50x for all samples across both platforms changed these values by <5%", how were the HiSeq X data increased to 50X? the author should provide more details as how the 50X genomes were generated from both platforms

    We mentioned this in the methods section. The original run from HiSeq X machine was 50X PCR-Free. We downsampled it to 35x to mimic the average coverage levels we observed in TopMed and CCDG, two of the larger cohort projects involving tens of thousands of genomes each.

    b. "The unfiltered concordance values, corresponding to all raw Parliament2 consensus calls, indicate low inter-platform consistency", the author should provide data to support the conclusion of 'low inter-platform consistency'

    We have included a figure (Figure 4) to show this more explicitly. Supplementary Table 2 gives more details.

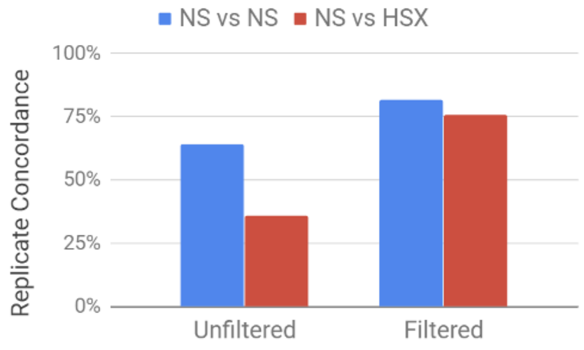

    c. "After filtering for Parliament2 events with a quality value greater than 3, inter- and intra-platform concordances increase to similar levels", how are 'similar levels' defined? How do the inter- and intra-platform concordance look like before and after filtering on quality value? Is quality value = consensus quality score ?

    We calibrated the quality score based on the lessons learned from the GIAB benchmark. The comparisons are done over SURVIVOR merge to compare the overlap of SV between the individual call sets. Detail results can be found in Supplementary Table 2 where we list the precision and recall values.

9. "1000 Genomes Project SVs for GRCh38"
    a. "The 1000 Genomes Project (1KGP) is a valuable resource of high-confidence SV calls across a large sample set (2,691 samples) mapped to GRCh37", the corresponding studies should have been cited; and which publications are the author talking about here? In Sudmant et al , SVs were called from 2504 genomes, where are the additional 187 samples from? The author should

provide more information as what samples and data are used here;

The samples were all previously remapped to GRCH38 and reported in the GigaScience publication. We cited the work as [22].

b. "Although the 1KGP samples have been remapped to GRCh38 [20,21], we are not aware of a comprehensive set of SVs on these data and reference sets". The Sudmant et al 2015 did provide SV calls on GRCH38

That is incorrect. Sudmant reported calls to GRCH37/hg19. From their paper in the methods: "We mapped Illumina WGS data (~100 bp reads, mean 7.4-fold coverage) from 2,504 individuals onto an amended version 8 of the GRCh37 reference assembly using two independent mapping algorithms—BWA17 and mrsFAST18—and performed SV discovery and genotyping using an ensemble of nine different algorithms (Extended Data Fig. 1 and Supplementary Note)."

c. Did the author used the low coverage (4-7X) 1000 genomes samples for the comparison? If so, why do the author use the cost of "running GATK4 on 220 WGS samples at 35x coverage" as reference? I cannot see any reason that they are comparable. And the coverage of the data should be explicated;

Correct, these samples were included. We used this comparison to again illustrate how inexpensive and efficient Parliament2 is relative to a workflow investigators in the field are likely to be familiar with. All the samples vary in coverage.

d. According to figure5, there are 400-500 SVs per sample that passed the filter of Parliament2, however, the 1000 genome phase 3 (Sudmant et al. 2015) callset represents ~4400 SVs per genome, and the more recent gnomadV2 callset estimated ~7400 SVs per genome. Compared against these studies, the estimated sensitivity of Parliament2 would be 5-10%, which is significantly different from what were described in the manuscript. The author should clarify the difference here.

We thank the reviewer for highlighting that because, in retrospect, this plot is indeed confusing. We tried to illustrate the shared amount of SV within the subpopulation.
*On average, across all samples, we identify 4,699.99 SVs on the passed Parliament2 calls. This is based on the low-coverage samples without genotyping the SVs and agrees with the highly-curated SV calls from the 1000 Genomes project. Most of these SVs (~3647.59) are deletions across these samples followed by other types, which is in concordance with the expectations from other studies. We have now replaced the plot showing the number of SV across the individuals as a whisker plot and the allele frequency of all SV across the population. Both follow the expectations. Nevertheless, the insertion detection rate is reduced as expected compared to deletions. This is also now highlighted better in HG002 and the other samples that we included for benchmarking (see **Supplementary Figure 2**).*

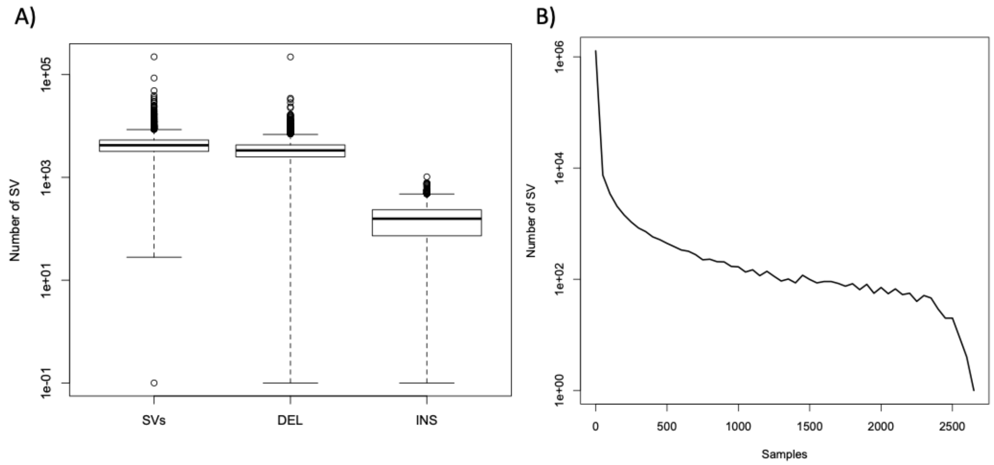

10. Page 12. Are these discussions? If so, they shall go under section "Discussion"

Yes, it is closing remarks/ discussions. However, the journal style says everything should be described in Findings: https://academic.oup.com/gigascience/pages/technical_note

11. Figure 3, panel B: legend truncated;
Thank you for pointing out the formatting issue. We have fixed it.

12. Where is Figure4?

Thank you for highlighting this. It must have been deleted over the last formatting step. We have now included it again.

13. Reference formatting:

We are sorry for this. This was caused by Paperpile, the reference manager we used.

a. What does [internet] mean?

   This was caused by a bug of the citation manager. Thank you for pointing this out.

b. Why are names of journals spelled in full for some and in abbreviation in others?

   This was caused by a bug of the citation manager. Thank you for pointing this out.

c. Ref 19 were not formatted correctly. Biorxiv preprints should be properly cited.

   Again, the citation manager. We have updated the citation as the paper was published.

~~~~~~

Reviewer #2: The authors present a new method for executing and merging multiple SV callers into a single VCF. The problem is important, the paper is well written, and the performance of the method is impressive, but I have a 2 major issues.
~~~~~~

We thank the reviewer for this recognition and comments.

There needs to be more details about what the ruleset is and how was generated, and the method needs to be tested against other genomes. The current manuscript just says it was based on GIAB deletions results, which means the samples was HG002. My concern is that you have essentially tuned Parliament2's performance on the same truth set that you test other methods against, which could possibly give us an overly optimistic view of this method's efficacy on unseen data. Even though you demonstrate this method's inter-platform concordance, this only partially assuages my concern here. The issue can be easily fixed by testing the methods from the paper against a few more samples with an existing truth set.
For example, Chaisson 2019 in Nat Com has SV call sets from three individuals that can serve as another gold standard to test against. A favorable comparison against these samples would make this result a lot stronger. (HG033!?)

We have now benchmarked Parliament2 across the 3 individuals from the Chaisson et al paper. Overall the benchmarks support the findings over HG002. We identified the highest recall and lowest false positive of Parliament2 compared to e.g. Manta and Delly. We further noticed a lower recall of insertions vs. depletion which is expected for short reads. For insertions, Parliament2 still shows the highest recall followed by Manta and a high precision. We have added the results as Supplementary Figure 3.

The authors totally missed FusorSV (Becker 2018, Genome Biology) which included a very similar set of SV tools, but used merge strategy that had was based on a similar observations (that different callers do better on different types and sizes), but FusorSV has an arguably more sophisticated method. I would also like to see this included in Figs 1 and 2 to know how Parliament2 stacks up to the ML machinery that FusorSV brings. (last commit Oct. 2019)

FusorSV is quite different from Parliament2. Parliament2 also manages and optimizes the execution of the SV callers enabling a SV calling at scale, whereas FusorSV focuses more on the merging of SV, similar to SURVIVOR. Regardless, we tried to benchmark FusorSV, but were unable to get it to run correctly. We have included MetaSV which, similar to FusorSV, focuses on the merging and requires the user to handle the SV calling given a list of programs.
