# Supplementary material for: Parliament2: Accurate structural variant calling at scale
Source: Gigascience. 2020 Dec 21;9(12):giaa145. doi: 10.1093/gigascience/giaa145 (PMC7751401; doi:10.1093/gigascience/giaa145)
Supplement: giaa145_Supplemental_Files [file giaa145_supplemental_files.zip › Figures_Parliament2.pptx]

## Slide 1
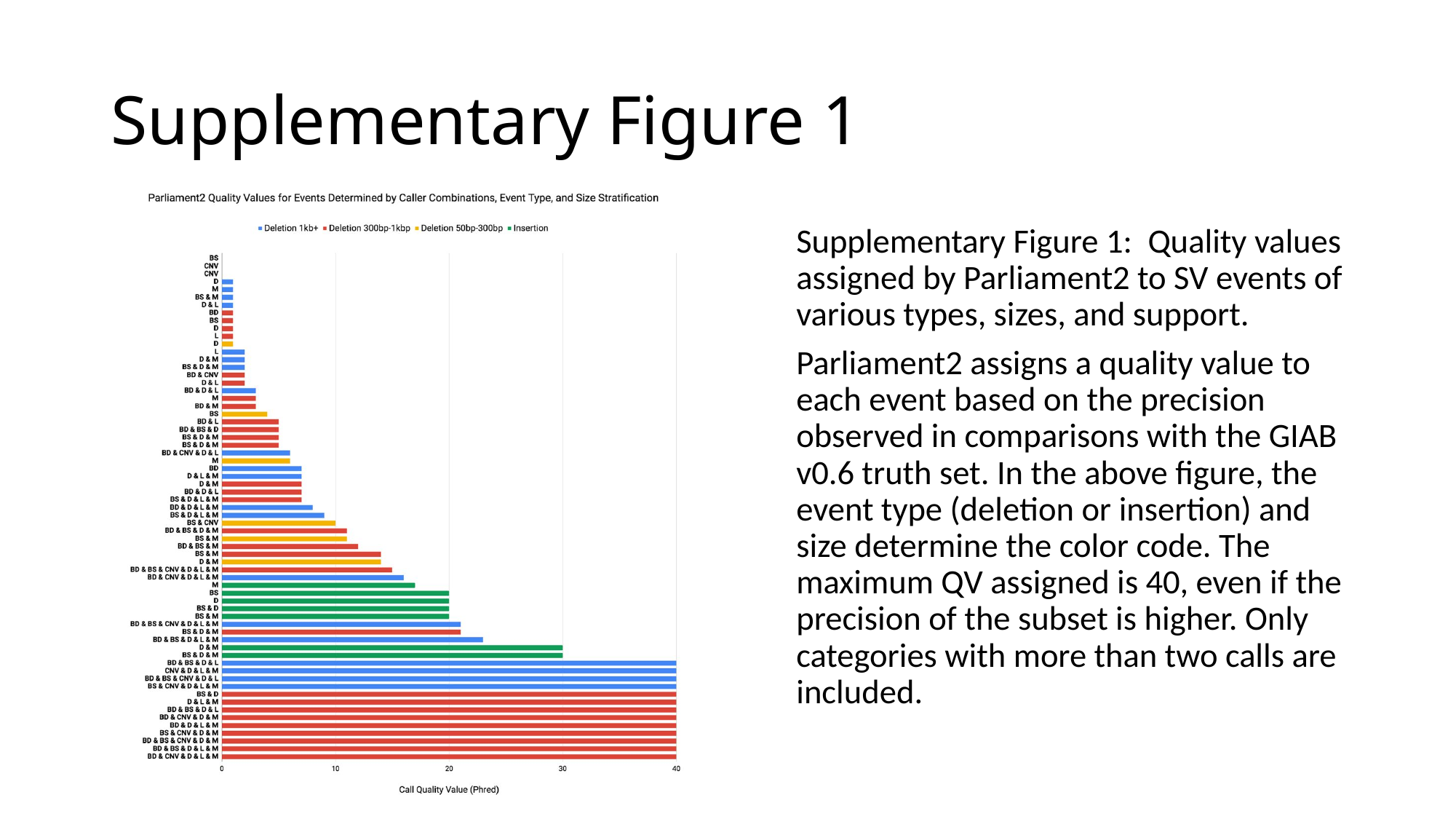

# Supplementary Figure 1
Supplementary Figure 1: Quality values assigned by Parliament2 to SV events of various types, sizes, and support.
Parliament2 assigns a quality value to each event based on the precision observed in comparisons with the GIAB v0.6 truth set. In the above figure, the event type (deletion or insertion) and size determine the color code. The maximum QV assigned is 40, even if the precision of the subset is higher. Only categories with more than two calls are included.

## Slide 2
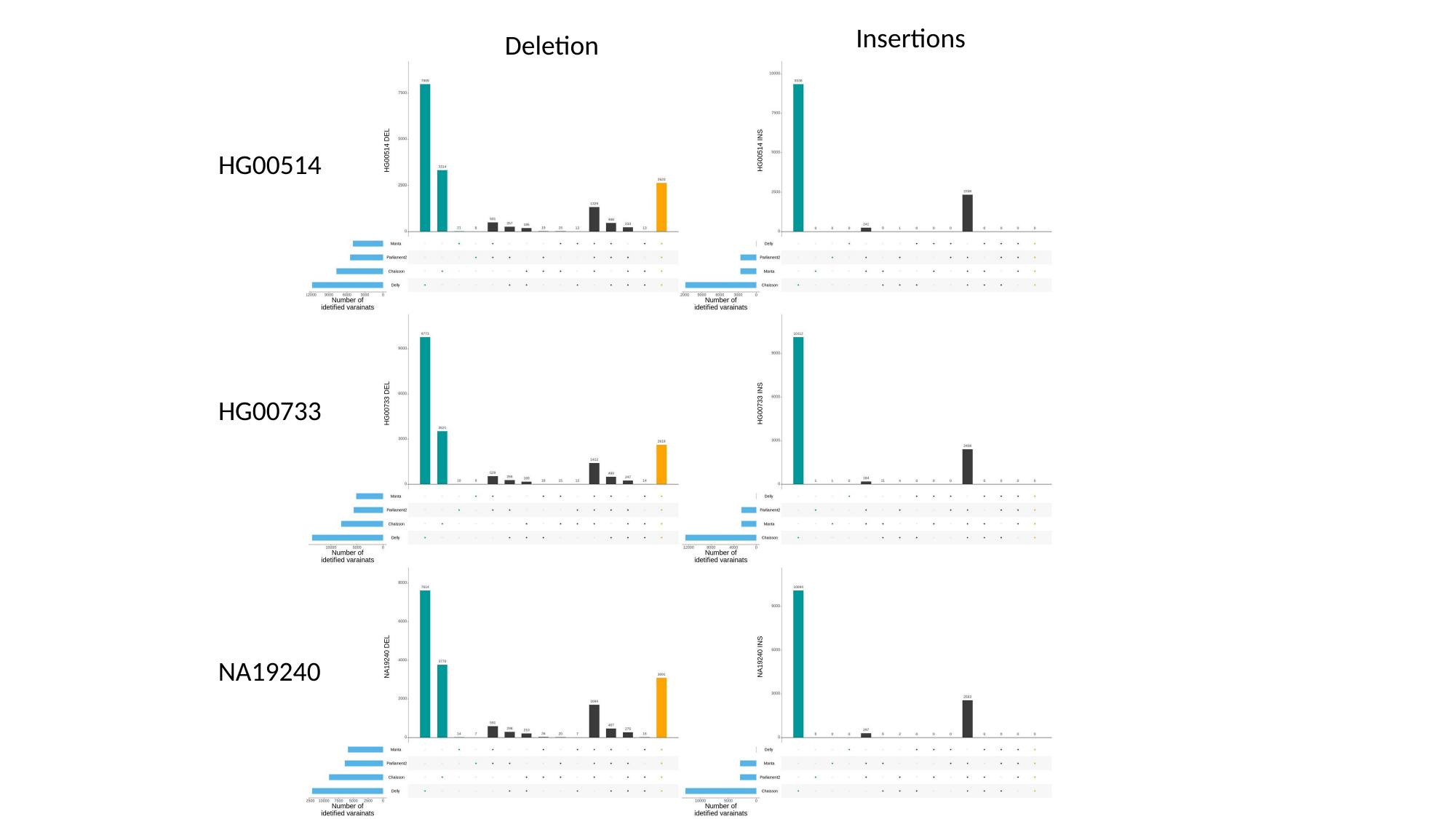

Insertions
Deletion
HG00514
HG00733
NA19240
