# Supplementary material for: Parliament2: Accurate structural variant calling at scale
Source: Gigascience. 2020 Dec 21;9(12):giaa145. doi: 10.1093/gigascience/giaa145 (PMC7751401; doi:10.1093/gigascience/giaa145)
Supplement: giaa145_Response_to_Reviewer_Comments_Original_Submission [file giaa145_response_to_reviewer_comments_original_submission.pdf]

Author's Response To Reviewer Comments

Close

We have attached the responses as the cover letter in the submission. The response also includes two new figures that would be lost in here.

Close
