# Supplementary material for: Parliament2: Accurate structural variant calling at scale
Source: Gigascience. 2020 Dec 21;9(12):giaa145. doi: 10.1093/gigascience/giaa145 (PMC7751401; doi:10.1093/gigascience/giaa145)
Supplement: giaa145_Response_to_Reviewer_Comments_Revision_1 [file giaa145_response_to_reviewer_comments_revision_1.pdf]

Author's Response To Reviewer Comments

Close

We have added the figures as separate files, included the GIGADB citation and point the reader to just one github repository now.

We thank the reviewer 1 to point us to the FTP. However, the README in the FTP states that this is a liftover and not a call set for SV. Thus, our point remains.

Close
