# Supplementary material for: Parliament2: Accurate structural variant calling at scale
Source: Gigascience. 2020 Dec 21;9(12):giaa145. doi: 10.1093/gigascience/giaa145 (PMC7751401; doi:10.1093/gigascience/giaa145)
Supplement: giaa145_Reviewer_1_Report_Original_Submission — Xuefang Zhao -- 4/23/2020 Reviewed [file giaa145_reviewer_1_report_original_submission.pdf]

Reviewer Report

Title: Parliament2: Accurate Structural Variant Calling At Scale

Version: Original Submission **Date:** 4/23/2020

Reviewer name: Xuefang Zhao

Reviewer Comments to Author:

Parliament2
Overall
This manuscript represents an cloud-based ensemble method that incorporate multiple state-of-the-art algorithms for SV discovery at high sensitivity, and implemented a series of quality control(QC) steps to ensure the specificity. Improved performance were observed when compared against individual algorithms, at the cost of higher computing costs / resources. While informative and timely, there are major flaws in the study design, such as wrong assumptions being adopted while calculating quality score, or conflict information delivered by the text and Figure 5. Moreover, several places in this manuscript deliver confusing information, and the author could use more help in professional writing.
Major:
1. Conclusion in the Abstract (page2):
Is Parlimament2 designed for SV discovery of single sample, or for group of samples?
2. Last sentence of the first paragraph under "Findings" (page2)
"Even best-in-class methods can fail to capture the majority of SVs (30% to 70% sensitivity) and often return a high false discovery rate, especially for insertion and inversion events [5,6]."
a. The author should define 'high false positive rate', by showing the actual FDR rate in this sentence
b. The author should also define 'best-in-class methods' , which specific algorithms are referred to here?
c. The two publications cited here both focused on SV discovery from long reads whole genome sequencing technology, which is a different sequencing platform and is not really comparable to any short read paired end sequencing methods. moreover, neither of the cited paper mentioned benchmark results of SVs from short read sequencing methods against long read methods, I don't think these two publications can be cited to support this sentence.
3. First sentence of the second paragraph under "Findings" (page2)
"Common SV detection methods, including Breakdancer [7], CNVnator [8], Crest [9], Delly [10], Lumpy [11],
Manta [11,12], and Pindel [13],"
'Common SV detection methods' reads confusing here, it can be interpret as either 'commonly used SV detection methods' or 'SV methods used to detect common SVs among population', the author should clarify;
4. Second paragraph on page3
"Parliament2 executes any combination of Breakdancer, Breakseq, CNVnator, Delly, Lumpy, and Manta to generate candidate SV events"

Does Parliament2 support any other algorithms? If yes, how? If no, why isn't any MEI specific algorithms included? None of these listed algorithms have shown comparable performance to MELT, it should be supported for a full scale SV discovery.

5. The section "Accuracy assessments for Parliament2 based on real data"

a. A brief description of the GIAB sample should be described here, as the authors should not expect audiences to have read the Zook et al 2019.

b. Parliament2 outputs SVs of different types, including deletions, duplications, insertions, inversions and translocations, however, only deletions are benchmarked. The author should also have provided the performance comparison of insertions. Focusing on deletions could be biased for algorithms that are specifically designed for deletions.

c. Why are lumpy and Breakdancer not included in comparison of deletions <300bp? Both methods should have generated good amount of deletions in this size range.

d. The author briefly described computational cost of the Parliament2 in one sentence: "Parliament2 ran in 3.43 hours (wall time) on a 16-core machine from a 35x coverage BAM aligned to the hs37d5 reference genome." But I don't see why this is necessary as there's a whole paragraph discussing the computational cost right after this paragraph. Moreover, this sentence is confusing, is only one sample used for the comparison here? Or is 3.43 hours averaged across multiple samples? If so, how many? The authors should have provided more information about the samples used for this comparison

e. Define F1 score, and explain why how does this score represents the performance of each methods;

6. "Compute Efficiency", page 6

a. The author should have provided estimation of the overall run time of Parliament2 on a 35X genome.

b. Direct comparison of computing cost in terms of overall CPU hours on fixed number of cores should be provided between Parliament2 and other individual methods. The author indicated better usage of computing source were achieved through parallelization, however the overall cost of running multiple algorithms, intergrade and quality control should still be higher than each individual algorithms. The comparison of overall cost can get audience a clear idea of the tradeoff between computing cost and increased performance when deciding on SV calling methods.

7. "Consensus Quality Scores", page 6

a. "One oft-discussed problem for short-read based SV calling is low sensitivity and high false discovery rates [5,6]." The author commented on the performance of current short read based SV discovery method, by citing two publications that focused on SV discovery from single molecule long reads sequencing technologies. However, Parliament2 is also a short read based method, so I wouldn't expect it to overcome all the challenges of short reads and achieve comparable performance to long reads, neither are there any such evidences provided in this manuscript. So I cannot see why the two publications were cited here and how they can be used to support the point.

b. "Based on these observations, we generated a ruleset based on GIAB deletion calls assuming the individual SV callers show similar metrics in other types of SVs." This assumption is not true, each type of SV should be analyzed independently.

c. If GIAB callset cannot provide enough benchmark data to derive consensus quality scores for SV types other than deletions and insertions(as the author stated, "The same ruleset is also applied to other SV types for which we lacked GIAB benchmark data (e.g. inversions)."), the author should seek

studies for gold standard SV callset such as Chaisson et al(2019, Nat Commun), which provided SV calls across different types, including inversions.

8. "Inter-Platform Concordance"

a. "Increasing coverage to 50x for all samples across both platforms changed these values by <5%", how were the HiSeq X data increased to 50X? the author should provide more details as how the 50X genomes were generated from both platforms

b. "The unfiltered concordance values, corresponding to all raw Parliament2 consensus calls, indicate low inter-platform consistency", the author should provide data to support the conclusion of 'low inter-platform consistency'

c. "After filtering for Parliament2 events with a quality value greater than 3, inter- and intra-platform concordances increase to similar levels", how are 'similar levels' defined? How do the inter- and intra-platform concordance look like before and after filtering on quality value? Is quality value = consensus quality score ?

9. "1000 Genomes Project SVs for GRCh38"

a. "The 1000 Genomes Project (1KGP) is a valuable resource of high-confidence SV calls across a large sample set (2,691 samples) mapped to GRCh37", the corresponding studies should have been cited; and which publications are the author talking about here? In Sudmant et al , SVs were called from 2504 genomes, where are the additional 187 samples from? The author should provide more information as what samples and data are used here;

b. "Although the 1KGP samples have been remapped to GRCh38 [20,21], we are not aware of a comprehensive set of SVs on these data and reference sets". The Sudmant et al 2015 did provide SV calls on GRCH38

c. Did the author used the low coverage (4-7X) 1000 genomes samples for the comparison? If so, why do the author use the cost of "running GATK4 on 220 WGS samples at 35x coverage" as reference? I cannot see any reason that they are comparable. And the coverage of the data should be explicated;

d. According to figure5, there are 400-500 SVs per sample that passed the filter of Parliament2, however, the 1000 genome phase 3 (Sudmant et al. 2015) callset represents ~4400 SVs per genome, and the more recent gnomadV2 callset estimated ~7400 SVs per genome. Compared against these studies, the estimated sensitivity of Parliament2 would be 5-10%, which is significantly different from what were described in the manuscript. The author should clarify the difference here.

10. Page 12. Are these discussions? If so, they shall go under section "Discussion"

11. Figure 3, panel B: legend truncated;

12. Where is Figure4?

13. Reference formatting:

a. What does [internet] mean?

b. Why are names of journals spelled in full for some and in abbreviation in others?

c. Ref 19 were not formatted correctly. Biorxiv preprints should be properly cited.

Level of Interest

Please indicate how interesting you found the manuscript: Choose an item.

Quality of Written English

Please indicate the quality of language in the manuscript: Choose an item.

Declaration of Competing Interests

Please complete a declaration of competing interests, considering the following questions:

- Have you in the past five years received reimbursements, fees, funding, or salary from an organisation that may in any way gain or lose financially from the publication of this manuscript, either now or in the future?
- Do you hold any stocks or shares in an organisation that may in any way gain or lose financially from the publication of this manuscript, either now or in the future?
- Do you hold or are you currently applying for any patents relating to the content of the manuscript?
- Have you received reimbursements, fees, funding, or salary from an organization that holds or has applied for patents relating to the content of the manuscript?
- Do you have any other financial competing interests?
- Do you have any non-financial competing interests in relation to this paper?

If you can answer no to all of the above, write 'I declare that I have no competing interests' below. If your reply is yes to any, please give details below.

I declare that I have no competing interests

I agree to the open peer review policy of the journal. I understand that my name will be included on my report to the authors and, if the manuscript is accepted for publication, my named report including any attachments I upload will be posted on the website along with the authors' responses. I agree for my report to be made available under an Open Access Creative Commons CC-BY license (http://creativecommons.org/licenses/by/4.0/). I understand that any comments which I do not wish to be included in my named report can be included as confidential comments to the editors, which will not be published.

Choose an item.

To further support our reviewers, we have joined with Publons, where you can gain additional credit to further highlight your hard work (see: https://publons.com/journal/530/gigascience). On publication of this paper, your review will be automatically added to Publons, you can then choose whether or not to claim your Publons credit. I understand this statement.

Yes Choose an item.
