# Supplementary material for: Parliament2: Accurate structural variant calling at scale
Source: Gigascience. 2020 Dec 21;9(12):giaa145. doi: 10.1093/gigascience/giaa145 (PMC7751401; doi:10.1093/gigascience/giaa145)
Supplement: giaa145_Reviewer_2_Report_Original_Submission — Ryan Layer -- 5/9/2020 Reviewed [file giaa145_reviewer_2_report_original_submission.pdf]

Reviewer Report

Title: Parliament2: Accurate Structural Variant Calling At Scale

Version: Original Submission **Date:** 5/9/2020

Reviewer name: Ryan Layer

Reviewer Comments to Author:

The authors present a new method for executing and merging multiple SV callers into a single VCF. The problem is important, the paper is well written, and the performance of the method is impressive, but I have a 2 major issues.
There needs to be more details about what the ruleset is and how was generated, and the method needs to be tested against other genomes. The current manuscript just says it was based on GIAB deletions results, which means the samples was HG002. My concern is that you have essentially tuned Parliament2's performance on the same truth set that you test other methods against, which could possibly give us an overly optimistic view of this method's efficacy on unseen data. Even though you demonstrate this method's inter-platform concordance, this only partially assuages my concern here. The issue can be easily fixed by testing the methods from the paper against a few more samples with an existing truth set.
For example, Chaisson 2019 in Nat Com has SV call sets from three individuals that can serve as another gold standard to test against. A favorable comparison against these samples would make this result a lot stronger.
The authors totally missed FusorSV (Becker 2018, Genome Biology) which included a very similar set of SV tools, but used merge strategy that had was based on a similar observations (that different callers do better on different types and sizes), but FusorSV has an arguably more sophisticated method. I would also like to see this included in Figs 1 and 2 to know how Parliament2 stacks up to the ML machinery that FusorSV brings.

Level of Interest

Please indicate how interesting you found the manuscript: Choose an item.

Quality of Written English

Please indicate the quality of language in the manuscript: Choose an item.

Declaration of Competing Interests

Please complete a declaration of competing interests, considering the following questions:

- Have you in the past five years received reimbursements, fees, funding, or salary from an organisation that may in any way gain or lose financially from the publication of this manuscript, either now or in the future?
- Do you hold any stocks or shares in an organisation that may in any way gain or lose financially from the publication of this manuscript, either now or in the future?
- Do you hold or are you currently applying for any patents relating to the content of the manuscript?
- Have you received reimbursements, fees, funding, or salary from an organization that holds or has applied for patents relating to the content of the manuscript?
- Do you have any other financial competing interests?
- Do you have any non-financial competing interests in relation to this paper?

If you can answer no to all of the above, write 'I declare that I have no competing interests' below. If your reply is yes to any, please give details below.

I declare that I have no competing interests

I agree to the open peer review policy of the journal. I understand that my name will be included on my report to the authors and, if the manuscript is accepted for publication, my named report including any attachments I upload will be posted on the website along with the authors' responses. I agree for my report to be made available under an Open Access Creative Commons CC-BY license (http://creativecommons.org/licenses/by/4.0/). I understand that any comments which I do not wish to be included in my named report can be included as confidential comments to the editors, which will not be published.

Choose an item.

To further support our reviewers, we have joined with Publons, where you can gain additional credit to further highlight your hard work (see: https://publons.com/journal/530/gigascience). On publication of this paper, your review will be automatically added to Publons, you can then choose whether or not to claim your Publons credit. I understand this statement.

Yes Choose an item.
